# Level of agreement between objectively determined body composition and perceived body image in 6- to 8-year-old South African children: The Body Composition–Isotope Technique study

**DOI:** 10.1371/journal.pone.0237399

**Published:** 2020-08-10

**Authors:** Lynn T. Moeng-Mahlangu, Makama A. Monyeki, John J. Reilly, Zandile J. Mchiza, Thabisile Moleah, Cornelia U. Loechl, Herculina S. Kruger

**Affiliations:** 1 Physical Activity, Sport and Recreation Research Focus Area, Faculty of Health Sciences, North-West University, Potchefstroom, South Africa; 2 Physical Activity for Health Group, School of Psychological Sciences and Health, University of Strathclyde, Glasgow, United Kingdom; 3 School of Public Health (SoPH), University of the Western Cape, Cape Town, South Africa; 4 International Atomic Energy Agency (IAEA), Division of Human Health, Vienna International Centre, Vienna, Austria; 5 Centre of Excellence for Nutrition, North-West University, Potchefstroom, South Africa; Kennesaw State University, UNITED STATES

## Abstract

To assess the level of agreement between body size self-perception and actual body size determined by body mass index (BMI) z-score and body fatness measured by the deuterium dilution method (DDM) in South African children aged 6–8 years. A cross-sectional sample of 202 children (83 boys and 119 girls) aged 6–8 years from the Body Composition–Isotope Technique study (BC–IT) was taken. Subjective measures of body image (silhouettes) were compared with the objective measures of BMI z-score and body fatness measured by the DDM. The World Health Organization BMI z-scores were used to classify the children as underweight, normal, overweight, or obese. DDM-measured fatness was classified based on the McCarthy centile curves set at 2nd, 85th and 95th in conjunction with fatness cut-off points of 25% in boys and 30% in girls. Data were analyzed using SPSS v26. Of 202 children, 32.2%, 55.1%, 8.8%, and 2.4% perceived their body size as underweight, normal, overweight, and obese, respectively. Based on BMI z-score, 18.8%, 72.8%, 6.9%, and 1.5% were classified as underweight, normal, overweight, and obese, respectively. Body fatness measurement showed that 2.5%, 48.0%, 21.8%, and 29.7% were underweight, normal weight, overweight, and obese, respectively. The application of silhouettes and BMI z-scores resulted in either overestimation or underestimation of own body size. Overall, the levels of agreements (kappa, κ) between body size perception, body fatness, and BMI for age respectively, were small (κ = 0.083, p = 0.053 and κ = 0.154, p<0.001). Level of agreement between body size perception, body fatness, and BMI z-score was poor. The use of silhouettes made children either overestimate their own body size while being underweight or underestimate their own body size while being overweight or obese. Given the potential health implications associated with misclassification of body size during childhood, correct self-assessment of body size is important, and may be key to the adoption of weight control strategies directed at curbing the escalating obesity epidemic in the country. Scalable measures to allow for more accurate self-assessment are urgently needed–one approach is behavior change communication at all levels.

## Introduction

Childhood obesity is becoming a global public health problem [1]. Being overweight or obese has been linked with a number of childhood metabolic conditions such as insulin resistance and cardiovascular disease [1–3]. Early identification of overweight and obese children should be prioritized in public health initiatives. Epidemiological studies highlight obesity as caused by a myriad of factors, including social and cultural influences [2, 4], and the consumption of energy-dense diets with low nutritional benefits [5]; ultra-processed foods including refined carbohydrates and added sugar; and foods high in sodium, saturated fat, and trans fats [6].

The first South African National Health and Nutrition Examination Survey (SANHANES-1), a survey of a national representative sample of South Africans conducted in 2012, showed that the prevalence of overweight and obesity was lower among children aged 6–9 years (4.5% and 2.7%, respectively) than in children aged 2–5 years (17.5% and4.4%, respectively) [7]. South Africa, being a developing country, is experiencing a nutrition syndemic (i.e. the coexistence of underweight and obesity, coupled with micronutrient deficiencies) across the lifespan [8]. The nutrition syndemic in children is partially related to the development of the pediatric metabolic syndrome [9–11]. Furthermore, obesity has been linked to psychological disorders that manifest as low self-esteem and body size dissatisfaction [12].

A number of scholars [13–18] suggest that compared with body mass index (BMI) and other objective weight measures, body size perception may be a better predictor of behavior change and is associated with better body size management. As such, correct self-assessment of body size is important, and may be the key to the adoption of weight control strategies directed at curbing the escalating obesity epidemic in the country. Body image perception is defined as a multidimensional construct encompassing how individuals perceive, think, and feel about their body size [19, 20]. Links between body image perception and psychosocial problems, e.g. low self-esteem and depression, have been observed [21–24]. Some researchers state that body image perception is dynamic and changes at different stages of development [25]. Weight perception is reported to be highly influenced during the growth spurt, when significant changes occur to the child’s body [20]. Other authors have identified culture and ethnicity [4], as well as media platforms such as television programs, cartoons, and magazines [26, 27], as contributory factors to body image dissatisfaction. Among Africans, larger bodies have been considered desirable [14, 28–31]. It can be argued that if not addressed, satisfaction with an oversized body may promote the development of obesity-related non-communicable diseases.

In the SANHANES-1 study [7], 84.5% of adult South Africans were shown to have a largely distorted body image, with 45.3% highly dissatisfied with what they perceived their body size to be at the time when the survey was undertaken. The findings also showed that overweight and obese South Africans underestimated their body size and had desires to be thinner. A study conducted with 11- to 15-year-old South African children from Mpumalanga Province [31] showed that the majority of the girls (83.5%) were dissatisfied with their body size. In this case, the children desired to be thinner or fatter than what they perceived their body size to be. Additionally, girls who wanted to be fatter had significantly higher BMI (p<0.001) than the girls who wanted to be thinner [31]. Findings from a prospective cohort study involving 278 adolescents aged13 to 17 years by Assaad and colleagues [32] reported that 39.9% and 10.1% perceived themselves as obese and overweight, respectively. The study also reported a fair agreement (60.8%, kappa statistic [κ] = 0.319) between measured BMI percentile and self-perceived BMI.

Poor correlation between body size perception and BMI in children and adolescents has been reported by various scholars [31, 33–36]. Some attribute this to children and adolescents possibly being unable to perceive their body size accurately, especially when the image is used twice to select current status and ideal body size [20, 37]. Lombardo and colleagues argued that body size perception in children below the age of 8 years is inaccurate [38]. In another study by Descamps reported that almost one-third of adolescents misperceived their body weight, with girls being more likely than boys to have body size misperceptions (27.3% of boys vs 42.2% of girls) [39]. Body image in children is frequently determined by figural stimuli such as silhouettes [40, 41]. Most of these figure scales show figure contours and avoid showing race characteristics such as hair and skin color, making the instruments reliable, valid, and suitable for use among different ethnic groups [42].

To the best of our knowledge, no research has attempted to compare the level of agreement between body size perception and measured body fatness in 6- to 8-year-old children from African countries, especially South Africa. The main aim of this study, therefore, was to determine self-perceived body size among 6- to 8-year-old South African children and the level of agreement between perceived and actual body size (BMI forage), as well as body fatness objectively measured by the deuterium dilution method (DDM).

## Materials and methods

### Study design and participants

A cross-sectional design was used with a total of 202 children (83 boys, 41.1% and 119 girls, 58.9%) aged between 6 and 8 years with complete data on body image by questionnaire, drawn from a sample of 299 children from a larger study on body composition using an isotope technique (the Body Composition–Isotope Technique [BC–IT] study; 2018/2019). Five primary schools were randomly selected from a total of 26 primary schools in Tlokwe City in North West Province, South Africa. The sample size was calculated using Open Epi software, Version 3 [43], in which Fleis’s [44] formulae for cross-sectional studies were applied to determine the appropriate sample size for a power of 0.80 and α-level of 0.05 at a CI of 95%. The power calculation was based on the primary hypothesis of the larger study, of a negative association between excessive fatness and physical activity and the odds of having excessive percentage body fat (%BF) in the inactive group, from which it was found that a minimum sample size of 297 was needed, based on an expected prevalence of combined overweight/obesity of 20% and physical inactivity of 30% in the children. Every third child on each class list was selected to participate in the study, but only those with signed parental informed consent forms who personally agreed to participate were finally included. The BC–IT larger study examined the relationships between more complex (using a stable isotope method and Bioelectrical Impedance Analysis (BIA)) and more indirect measures of body composition (using anthropometric variables), and objective and subjective measures of physical activity, and their relationships thereof, with other health-related determinants, among 6- to 8-year-old South African children. The parents of the children in the study as well as the children gave consent and assent to participate. The school principals and the district office of the Department of Basic Education in Dr KKKaunda District of North West Province granted study permission. Subsequently, the Health Research Ethics Committee of the Faculty of Health Sciences (HREC) of North-West University gave ethical approval for the study (ethics no: NWU-00025-17-A1).

### Anthropometry

Weight was measured with a portable electronic scale (Beurer Ps07 Electronic Scale, Ulm, Germany) to the nearest 0.1 kg and children were measured wearing light clothing, without shoes on. Stature was measured to the nearest 0.1 cm using a Harpenden portable stadiometer (Holtain Limited, UK) with a perpendicular board. BMI was calculated as weight divided by height squared (weight in kg/height squared in meters) and BMI z-scores were calculated relative to World Health Organization (WHO) reference data. The WHO BMI z-score categories used were: underweight:–2 SD from the median; normal weight: –2 SD to +1 SD; overweight: more than +1 to +2 SD; and obesity: more than +2 SD [45].

### Body fat percentage by stable isotope dilution

The DDM and protocol used in our study followed the guidance provided by the International Atomic Energy Agency (IAEA) [46, 47] and were also implemented as part of a multicenter study involving eight other African countries [48]. Pre-dose saliva samples were obtained from the study children after an overnight fast. Then, each child was given a dose of accurately weighed deuterium oxide-labelled water, which was prepared according to their body weight. Doses were administered under close supervision, with drinking straws to avoid potential spillage. Each child’s dose bottle was rinsed twice with water (50 ml per rinse) and the rinsing water was consumed to ensure complete intake of the dose. The time of dose administration was recorded for each child. Two saliva samples were collected, at 2 hours and 3 hours post dose. From the time of dose administration until the last saliva sample was collected, children were asked to stay in the study location and were required to avoid physical activity, to minimize water loss in breath and evaporation from the skin. Children had access to non-vigorous activities such as coloring books and storytelling to prevent boredom during the waiting period until the study protocol was completed. Children were not allowed to eat or drink anything apart from the light snack that was provided after completion of the sample collection, but they were permitted to go to the bathroom to void their bladders at any time. At each saliva sampling, the child was given a ball of cotton wool to rotate in the mouth until well soaked; the wet cotton wool was then transferred into a syringe to squeeze out the saliva into a plastic vial that was immediately capped to avoid evaporation [41]. For children who could not wet their cotton wool balls after several attempts, an alternative approach was used; such children were asked to spit directly into the vial. The plastic vial was encased in a sealable bag preventing possible losses from evaporation and cross-contamination during storage. Saliva samples were stored at –20°C in the laboratory until the analysis was performed. Saliva samples were analyzed using Fourier transform infrared (FTIR) spectroscopy (FTIR 4500t spectrophotometer, Agilent). Fat free mass (FFM) was calculated using age- and gender-specific Lohman hydration factors for children [49, 47]. Fat mass (FM) was calculated by the difference between body mass and FFM: FM (kg) = body mass (kg)–FFM (kg) [50]. %BF was calculated as FM (kg) / body mass (kg) × 100. Children were classified as under fat, overeat, and obese at the 2^nd^, 85^th^, and 95^th^ centiles based on the graphs of McCarthy et al. [51] for Caucasian children. Above 9^th^ to below 85^th^centilewas considered as normal fatness for this study. However, for the categories of overweight and obese the cut-off points of 25% body fat in boys and 30% body fat in girls by Williams et al. [52] were applied, which had been established using a skinfold thickness method previously validated against a multicomponent model to measure body fatness. The Williams [52] cut-off points, though relevant for African children, do not include under fat cut-off points. Therefore, the combination of the two sets of cut-off points would yield unbiased results. Additionally, the cut-off points by Williams [52] are grounded in the findings that excessive fatness has been linked with increased cardiometabolic risk profiles across wide age ranges [53].

### Body image silhouettes

A simple questionnaire adapted from another questionnaire that assessed body image status among girls and women [54] was used to assess body image perception, together with eight pictorial silhouettes of similar line drawings of girls and boys covering eight different sizes from thin to obese [55]. The girl silhouettes had previously been used in South African children [15, 31]. The eight silhouettes for boys had not been tested in South Africa among 6- to8-year-old boys. Self-perceived body size was assessed based on responses to one question: “Which of the images resembles your weight?” A figure scale linked to this question was presented and explained to each child by a well-trained research assistant, before a child made a choice of a figure that resembled him/her. The intra-class correlation between self-perceived body size and children’s actual BMI category was 0.36, with a Cronbach’s alpha value, based on standardized items, of 0.74. The body image perception of each child was compared with the BMI using the BMI z-scores and with the %BF objectively measured using the DDM.

The silhouettes used were from Stunkard Figure Ratings scales adapted for children [54]. The figure scales in our study were adapted to match the BMI classification for children older than 5 years as follows: underweight was shown to look like silhouettes A and B, normal weight like silhouettes C and D, overweight like silhouettes E and F, and obese like silhouettes G and H [42, 54, 55].

### Statistical analysis

Statistical analyses were performed using the Statistical Package for Social Sciences (SPSS v26.0). Descriptive statistics (mean, minimum, maximum, and SDs) and the *t*-test were used to compare groups. Pearson’s Chi-square (χ^2^) test was used to determine significant differences for the BMI, BMI z-scores, %BF, and body weight perception categories, separately by sex. The agreement between children’s own perceptions and actual body weight/fatness across the four categories was assessed using Cohen’s kappa (κ) statistic. Furthermore, agreement between children’s own perceptions across the four categories and actual body fatness was compared using Cohen kappa’s statistic (κ) to determine the magnitude of agreement between perceived and actual body size. Categories of actual body size (fatness) were derived for each child based on their %BF measured by the DDM. Additionally, the actual difference in percentages between the perceived body image and BMI, as well as the difference between perceived body weight and body fatness, were calculated. Weighted κ coefficients (criterion validity) were interpreted according to Landis and Koch for strength of agreement: κ <0.20: poor agreement; κ = 0.21–0.40:fair agreement; κ = 0.41–0.60: moderate agreement; κ = 0.61–0.80: good agreement; and κ = 0.81–100: perfect/very good agreement [56]. Kendall's tau-c (*T*_c_, also called Stuart-Kendall tau-c), a non-parametric measure, was used to assess the concordance among the children’s body image perception silhouette choice, BMI, and %BF determined by DDM. *T*_c_ ranges from 0 (no agreement) to 1 (complete agreement) and is more suitable than tau-b for the analysis of data based on non-square (i.e. rectangular) contingency tables [57–59]. The level of statistical significance was set at p≤0.05.

## Results

Table 1 represents descriptive characteristics of boys and girls. No significant gender differences (p>0.05) were found with age, weight, height, BMI, and BMI for age z-score. Significant gender differences (p<0.001) were observed for FM, %BF, and FFM in kg, with girls having significantly greater FM and %BF (p<0.001) than boys. However, boys had significantly (p = 0.004) more FFM (17.36±2.74) than girls (16.21±2.85).

**Table 1:** Participant characteristics.

				Boys (n = 83)	Girls (n = 119)	P-value for gender differences
	Mean	Minimum	Maximum	Mean ±SD	Mean ±SD	
Age (years)	7.57±0.85	6.00	8.96	7.62±0.79	7.54±0.90	0.55
Weight (kg)	22.70±4.53	14.80	43.60	22.69±4.15	22.76±4.80	0.91
Height (cm)	120.21±7.02	103.50	139.90	120.83±6.25	119.76±7.50	0.28
FFM (DDM) (kg)	16.67±2.84	10.71	27.12	17.36±2.74	16.21±2.85	0.004
FM (DDM) (kg)	6.03±2.45	2.23	19.49	5.30±2.06	6.56±2.58	<0.001
%BF (DDM)	26.04±6.17	13.04	44.71	22.02±5.30	28.14±5.87	<0.001
BMI (kg/m^2^)	15.60±1.99	12.03	26.99	15.43±1.74	15.73±2.15	0.29
BMI for age z-score	–0.17±1.08	–2.92	3.25	–0.30±1.08	–0.10±1.07	0.18

%BF: Percentage body fat; BMI: body mass index; DDM: deuterium dilution method; FFM: fat free mass; FM: fat mass; SD: standard deviation.

The results in Table 2 reflect how children viewed themselves based on their body size perception, measured using silhouettes and compared with actual weight classification as determined by BMI and the DDM. Using χ^2^ for categorical variables, significant differences were found in %BF measured by the DDM and BMI for both boys and girls, and borderline significant differences in silhouettes chosen for ideal weight status for both genders.

**Table 2:** Perceived body sizes among children by total group and sex.

	Responses	Total group (N = 202)		Boys (n = 83)				Girls (n = 119)		χ^2^	p-value
		n	%	n	%	χ^2^	p-value	n	%		
**Silhouette chosen for ideal weight status**	Underweight	66	33.0	33	39.3	7.072	0.07	33	27.3	23.084	<0.0001
	Normal for your age	113	56.0	41	48.8			72	59.5		
	Overweight	18	9.0	6	7.1			12	9.9		
	Obese	5	2.0	3	3.6			2	1.7		
**%BF measured using DDM**	Under fat/thinness	5	2.5	5	6.0	51.024	<0.0001	-	-	5.765	0.056
	Normal	97	48.0	48	57.8			49	41.2		
	Overfat	42	20.8	14	16.9			28	23.5		
	Obese	58	28.7	16	19.3			42	35.3		
**BMI**	Underweight BMI z < –2 SD	38	19.0	19	22.9	113.964	<0.0001	19	16.0	146.714	<0.0001
	Normal BMI z –2 to +1 SD	147	73.0	61	73.5			86	72.3		
	Overweight BMI z > +1 SD	14	7.0	2	2.4			12	10.1		
	Obese BMI z > +2	3	1.5	1	1.2			2	1.7		

%BF: Percentage body fat; BMI: body mass index; DDM: deuterium dilution method.

χ^2^: Chi-square test to compare categories between boys and girls.

Table 3 shows that out of 202 children, 2.5% (n = 5), 48.0% (n = 97), 20.8% (n = 42), and 28.7% (n = 58) were underfat, normal, overfat, and obese, respectively, as determined by the DDM. When BMI z-scores were used, 19.0% (n = 38), 73% (n = 147), 7% (n = 14), and 1.0% (n = 3) were underweight, normal, overweight, and obese, respectively. Based on the silhouettes they selected, 33.0%(n = 66), 56.0% (n = 113), 9.0%(n = 18), and 2.4%(n = 5) perceived their body size as underweight, normal, overweight, and obese, respectively.

**Table 3:** Differences in actual absolute numbers and percentage for silhouettes, BMI, and %BF categories by DDM.

Classifications	DDM	Silhouettes	Differences in absolute number and percentage, n (%)
	n(%)	n(%)	n(%)
Underweight	5(2.5)	66(33)	61(92)*
Normal weight	97(48.0)	113(56)	16(14)*
Overweight	42(20.8)	18(9)	24(57)^#^
Obese	58(28.7)	5(2)	53(91)^#^
	**DDM**	**BMI**	
Underweight	5(2.5)	38(19)	33(87)*
Normal weight	97(48)	147(73)	50(34)*
Overweight	42(20.8)	14(7)	28(67)^#^
Obese	58(28.7)	3(1)	55(95)^#^
	**BMI**	**Silhouettes**	
Underweight	38(19)	66(33)	28(42)*
Normal weight	147(73)	113(56)	34(23)^#^
Overweight	14(7)	18(9)	4(22)*
Obese	3(1)	5(2)	2(40)*

%BF: Percentage body fat; BMI: body mass index; DDM: deuterium dilution method.

^*ǂǂ*^represents underestimation and

* represents overestimation.

On comparing the accuracy of body size identification between perception using silhouettes and the measures of body fatness using the DDM (Table 3), the silhouette method was shown to overestimate underweight by 92.0% and underestimate overweight and obesity by 57.0% and 91.0%, respectively. These findings suggest that measuring body size perception using silhouettes was 12 times more likely to overestimate underweight than using the DDM, while children were 2 and 11 times more likely to underestimate overweight and obesity, respectively, compared with the DDM. On comparing the accuracy of body size identification between perceptions using silhouettes and BMI, the silhouettes overestimated underweight, overweight, and obesity by 42.0%, 22.0%, and 40.0%, respectively, compared with BMI z-score category. Overall, using silhouettes for body size perception overestimates underweight and grossly underestimated obesity compared with the DDM, whilst comparison between silhouettes and BMI z-score category indicated that silhouettes overestimate underweight, overweight, and obesity.

In comparing the accuracy of body composition measurements between BMI z-score category and the DDM, BMI z-score category was seven times more likely than the DDM to overestimate underweight and more likely to underestimate overweight and obesity.

Table 4 presents categories of %BF using the DDM and κ level of agreement between the DDM and perception of body image by silhouettes. In the total group, 2 of the children who were actually underweight perceived themselves as underweight, 30 who were normal weight perceived themselves as normal weight, and 8 and 9 who were actually overweight and obese, respectively, perceived themselves as overweight or obese. The level of agreement between body weight perception when compared with the DDM was poor (κ = 0.083, p = 0.053) for the total group. The concordance determined by *T*_c_ between DDM and silhouette body image perception was low and significant (*T*_c_ = 0.154, p = 0.011) for the total group.

**Table 4:** Cohen’s κ for percentage of body fat measured by DDM and self-perception of weight using silhouettes.

Gender			Current weight silhouettes (count)				Total
			Underweight (Pictures 1 & 2, –3 SD to –1 SD)	Normal weight (Pictures 3 & 4, –1 SD to +1 SD)	Overweight (Pictures 5 & 6, > +1 SD)	Obesity (Pictures 7 & 8, > +2 SD)	
Males	%BF by DDM	Underfat	2	2	0	1	5
		Normal	17	10	13	8	48
		Overfat	8	3	1	2	4
		Obese	3	7	0	3	16
	Total		30	22	17	14	83
Females	%BF by DDM	Underfat	-	-	-	-	-
		Normal	22	20	7	0	49
		Overfat	10	9	7	2	28
		Obese	11	6	19	6	42
	Total		43	35	33	8	119
Total	%BF by DDM	Underfat	2	2	0	1	5
		Normal	39	30	20	8	97
		Overfat	18	12	8	4	42
		Obese	14	13	22	9	42
	Total		73	57	50	22	202
**Symmetric measures**						
Gender			Value	Asymptotic standard error^a^	Approximate T^b^	Approximate significance	
Males	Ordinal by ordinal	*T*_*c*_	.006	.090	.068	.946	
	Measure of agreement	κ	–.023	.058	–.382	.702	
	N of valid cases		83				
Females	Ordinal by ordinal	*T*_*c*_	.299	.075	3.940	.000	
	Measure of agreement	κ	.152	.063	2.530	.01	
	N of valid cases		119				
Total	Ordinal by ordinal	*T*_*c*_	.154	.061	2.541	.011	
	Measure of agreement	κ	.083	.045	1.933	.053	
	N of valid cases		202				

%BF: Percentage body fat; DDM: deuterium dilution method; *T*_c_: Kendall’s tau-c.

^a^ Not assuming the null hypothesis.

^b^ Using the asymptotic standard error assuming the null hypothesis.

When κ was calculated separately for boys and girls, the level of agreement for boys was poor (κ = 0.023, p = 0.702) compared with the girls (κ = 0.152, p<0.01). Twoof the boys who were actually underweight perceived themselves as underweight, 10 who actually had normal weight perceived themselves as normal, and 1 and 3, respectively, who were overweight and obese perceived themselves as overweight and obese. The concordance determined by *T*_c_ between DDM body fatness and silhouettes (perception of body image) in boys was very low and not significant (*T*_c_ = 0.006, p = 0.946). No girls were actually underweight and perceived themselves as underweight, 20 girls of actual normal weight perceived themselves as having normal weight, and 7 who were actually overweight and 6 actually obese perceived themselves as overweight and obese, respectively. The concordance determined by *T*_c_ between the DDM measurement and perception among the girls was fair (*T*_c_ = 0.299, p<0.001).

Table 5 presents BMI categories and κ level of agreement between BMI category and perception of body image by silhouettes. In the total group, 24 children who were actually underweight perceived themselves as underweight, 47 who were normal weight perceived themselves as normal weight, and 8 and 2, who were actually overweight or obese, respectively, perceived themselves as overweight or obese. The level of agreement between body weight perception when compared with BMI category was poor, although significant (κ = 0.154; p<0.001) for the total group. The concordance determined by *T*_*c*_ between BMI category and body image perception by silhouette was low but significant (*T*_c_ = 0.231, p<0.001) for the total group.

**Table 5:** Cohen’s κ for body weight by body mass index category and perception by silhouette.

BMI_Categ_ANALYSES * CURRENTWEIGHT_SILHOUETTES * SEX Cross tabulation							
Gender			Current weight silhouettes (count)				Total
			Underweight (Pictures 1 & 2, –3 SD to –1 SD)	Normal weight (Pictures 3 & 4, –1 SD to +1 SD)	Overweight (Pictures 5 & 6 >, +1 SD)	Obesity (Pictures 7 & 8, >+2 SD)	
Males	BMI	Underweight	9	3	3	4	19
		Normal	21	17	14	9	61
		Overweight	0	1	0	1	2
		Obese	0	1	0	0	1
	Total		30	22	17	14	83
Females	BMI	Underweight	15	4	0	0	19
		Normal	28	30	25	3	86
		Overweight	0	1	8	3	12
		Obese	0	0	0	2	2
	Total		43	35	33	8	119
Total	BMI	Underweight	24	7	3	4	38
		Normal	49	47	39	12	147
		Overweight	0	2	8	4	14
		Obese	0	1	0	2	3
	Total		73	57	50	22	202
**Symmetric measures**						
Gender			Value	Asymptotic standard error^a^	Approximate T^b^	Approximate significance	
Males	Ordinal by ordinal	*T*_*c*_	.053	.075	.704	.481	
	Measure of agreement	κ	.040	.052	.755	.450	
	N of valid cases				83				
Females		*T*_*c*_	.378	.055	6.857	.000	
	Measure of agreement	κ	.232	.056	4.888	.000	
	N of valid cases		119				
Total	Ordinal by ordinal	*T*_*c*_	.231	.048	4.791	.000	
	Measure of agreement	κ	.154	.040	4.342	.000	
	N of valid cases				202				

BMI: Body mass index; *T*_*c*_: Kendall’s tau-c.

^a^Not assuming the null hypothesis.

^b^Using the asymptotic standard error assuming the null hypothesis.

When κ was calculated separately for boys and girls, 9 of the boys who were actually underweight perceived themselves as underweight, 17 who were actually normal weight perceived themselves as normal weight, and none of the boys who were overweight and obese perceived themselves as overweight and obese. The level of agreement between body weight perception and BMI category was poor (κ = 0.040; p = 0.450). The concordance between BMI category and body image perception by silhouette was very low and not significant (*T*_*c*_ = 0.053, p = 0.481).

For the girls, 15 were actually underweight and perceived themselves as underweight, 30 who were normal weight perceived themselves as normal weight, and 8 who were overweight and 2 who were obese perceived themselves as overweight and obese, respectively. The level of agreement between body weight perception and BMI category was poor, but significant (*κ* = 0.232; p<0.001). The concordance between BMI category and perception among the girls was moderate and significant (*T*_*c*_ = 0.378, p<0.001).

## Discussion

The main purpose of the study was to assess the level of agreement between self-perceived body size, BMI category, and actual body fatness determined using DDM, in 6- to 8-year-old South African school-going children. Generally, a poor to fair level of agreement between self-perceived body size, actual body fatness, and BMI category was found among the children. In summary, self-perceived weight status was generally not consistent with actual weight status (BMI forage z-score) and objectively measured body fatness.

Studies of children that have used DDM to measure body compositionhave been limited from African countries, including South Africa. One example is a multi-country study that generated data on body composition from 1516 children aged 8 to 11 years from eight African countries (Ghana, Kenya, Mauritius, Morocco, Namibia, Senegal, Tunisia, and the United Republic of Tanzania). The prevalence of excessive fatness, measured using DDM, was three times higher (29.1%–31.4%, n = 441) than the prevalence of overweight or obesity (8.8%–10.1%, n = 133) defined as WHO BMI z-score > +2 SD [48].

In another study, undertaken in 9727 children aged 7 to18 years from four districts in the Jin City of China, the prevalence of underweight, normal, and overweight determined by BMI was 4.9%, 64.3%, and 30.8%, respectively, whilst approximately 19.8%, 57.8%, and 22.4%, perceived themselves as underweight, normal weight, and overweight, respectively. This study confirmed that underweight is overestimated and overweight underestimated when using body size perception as a measure [20]. The prevalence of underweight was much lower and the prevalence of overweight much higher than in our study. Many factors could have contributed to these differences. Ethnic differences in fat mass have been reported in previous studies [60]. The differences in the perception of body size in the two studies could be partly due to the use of different tools to assess body image. Wang and colleagues used questions to elicit information about children’s weight status [20], whereas in our study, body size perceptions were based on a scale of eight silhouettes, which has been validated for use in South African children [54].

In our study, children’s own body size perception using silhouettes was 4 times and 2 times more likely to overestimate underweight and 1.8 times and 3.8 times more likely to underestimate overweight and obesity, respectively, when compared with body fatness measured using the DDM. Our findings are somewhat similar to the findings from a study in Iran reporting that 40% of the children misperceived their body size by either overestimating underweight or underestimating obesity status [61]. In a study conducted in Lebanon using a sample of 278 adolescents aged 13–17 years, a third (30.5%) of the adolescents and more than half (56.8%) of the obese adolescents underestimated their weight [32]. In our study, BMI was compared with body fatness measured using the DDM and the results showed that children were 3 and 19 times more likely to underestimate overweight and obesity, respectively.

In Wang’s study [20], a significant poor consistency between BMI and body weight perception was reported (κ = 0.352, p<0.05), in 7- to 8-year-oldChinese children (N = 9727) from four districts of Jilin City. In our study, the consistency of agreement between actual fat ness as assessed using the DDM and perceived body size using silhouettes was very poor (κ = 0.083; p = 0.053), hence the low level of concordance between the silhouettes and the DDM. The validity of the girls’ perceived body weight compared with objectively measured body fatness in our study demonstrated a fair agreement, and this finding is different from previous studies on the body weight perceptions of children’s parents from Mexico [62] and South America [41]. A study from Chile [63] reported poor concordance between nutritional status, perceived with a seven-figure scale, and real BMI (κ = 0.031, *T*_c_ = 0.275), and their finding was similar to the one for boys in our study (κ = 0.040, *T*_c_ = 053). Interestingly, BMI category and body size perception in our study had moderate validity among overweight girls and the lowest validity among girls with obesity. This is congruent with a study conducted in Minneapolis in the United States among 9- to 18-year-old boys and girls, which indicated that overweight and obese children were more likely to underestimate their body sizes than children of normal weight [40].

The observed poor agreement between BMI category and body size perception measured by silhouettes in our study is similar to the findings of the Youth/Child Cardiovascular and Environmental (SAYCARE) multi-country study, carried out in seven South American cities (Buenos Aires, Lima, Medellín, Montevideo, Santiago, São Paulo, and Teresina) with 1035 children and adolescents aged 3 to 17 years, which reported that the correlation between children’s body size self-evaluation outcome and their objectively measured BMI was lower before the age of 8 years [41]. The potential methodological errors associated with using silhouette choice in younger children as a reason for low correlation has been highlighted [62, 64].

### Limitation and strengths

#### Strengths

The strength and uniqueness of this study is that it was the first study in South Africa where a triangulation in the level of agreement between actual fatness measured using the DDM, BMI category, and body image perception using silhouettes in children aged 6 to 8 years was conducted. Additionally, 202 South African children is a relatively large sample size with adequate power for using the DDM to measure body fat, further contributing to the strength of the study.

#### Limitations

Our study did not consider the perception of parents and environmental factors that could influence children’s body size perceptions. However, studying these parameters was beyond the study scope. The study did not look at the full spectrum of children within this development stage. Children were selected from a homogenous environment; and therefore, the results cannot be extrapolated to other ethnic groups. The body fatness cut-offs used were developed in European children, and future research should develop cut-off points for fatness that are valid in African populations. Another limitation of the study is the lack of validation studies on body size perception in comparison to BMI and body fatness in South Africa and internationally. As such, further studies are required to develop a common standard body size perception measurement tool for proper comparison.

## Conclusion

We conclude that children either overestimated being under weight or underestimated being normal weight, overweight, and obese when using silhouettes, compared with their actual measured outcomes, especially those that are measured using the DDM. Body size self-perception in South African children is generally inaccurate. Given the potential health implications associated with misclassification of body size during childhood, such as weight increase or obesity, correct self-assessment of body size is important, and may be the key to the adoption of weight control strategies directed at curbing the escalating obesity epidemic in the country. Scalable measures to allow for more accurate self-assessment are urgently needed–one approach is behavior change communication (BCC) at all levels. Demystifying “big is better” will be a good starting point.

## Supporting information

S1 Data BC—IT study data used for the manuscript. (XLSX)

S1 File Body image questionnaire boys and girls. (DOCX)

S2 File Protocol of body fatness. (PDF)
