# Level of agreement between objectively determined body composition and perceived body image in 6- to 8-year-old South African children: The Body Composition–Isotope Technique study

**DOI:** 10.1371/journal.pone.0246879

**Published:** 2021-02-04

**Authors:** Lynn T. Moeng-Mahlangu, Makama A. Monyeki, John J. Reilly, Zandile J. Mchiza, Thabisile Moleah, Cornelia U. Loechl, Herculina S. Kruger

The sentence “At each saliva sampling, the child was given a ball of cotton wool to rotate in the mouth until well soaked; the wet cotton wool was then transferred into a syringe to squeeze out the saliva into a plastic vial that was immediately capped to avoid evaporation [41]” should cite reference 46 instead of 41.

The correct sentence should read: At each saliva sampling, the child was given a ball of cotton wool to rotate in the mouth until well soaked; the wet cotton wool was then transferred into a syringe to squeeze out the saliva into a plastic vial that was immediately capped to avoid evaporation [46].

There is an error in reference 47. The correct reference is: International Atomic Energy Agency (IAEA). Introduction to Body Composition Assessment Using the Deuterium Dilution Technique with Analysis of Saliva Samples by Fourier Transform Infrared Spectrometry. Human Health Series No. 12. Vienna: IAEA; 2011.

## References

[1] Moeng-Mahlangu LT, Monyeki MA, Reilly JJ, Mchiza ZJ, Moleah T, Loechl CU, et al (2020) Level of agreement between objectively determined body composition and perceived body image in 6- to 8-year-old South African children: The Body Composition–Isotope Technique study. PLoS ONE 15(8): e0237399 10.1371/journal.pone.0237399 32777810 PMC7417193

