# Supplementary material for: Level of agreement between objectively determined body composition and perceived body image in 6- to 8-year-old South African children: The Body Composition–Isotope Technique study
Source: PLoS One. 2020 Aug 10;15(8):e0237399. doi: 10.1371/journal.pone.0237399 (PMC7417193; doi:10.1371/journal.pone.0237399)
Supplement: S1 File — (DOCX) [file pone.0237399.s002.docx]

**BC-IT: BODY IMAGE SILHOUETTES**

**BODY IMAGE QUESTIONNAIRE FOR GIRLS AND BOYS**

*The interviewer with explain the purpose of this questionnaire.*

**This questionnaire is about the way you feel about your appearance in relation to your weight.**

**Interviewer’s name/ID ____________child’s ID/ number__________**

**Date of interview ---------------------------**

**Age------------------------ Weight______Kg__ Height_______m**

**Sex M F**

1. **Do you have any concerns about your current Weight Yes No**

**Height Yes No**

**Body image silhouettes**

*Girls only*

*
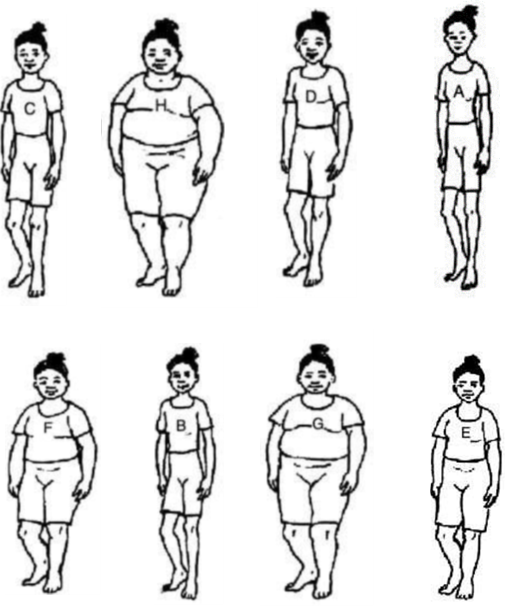
*

1. Which of the images resembles your current weight?

**________________________________________________________________**

**Boys**


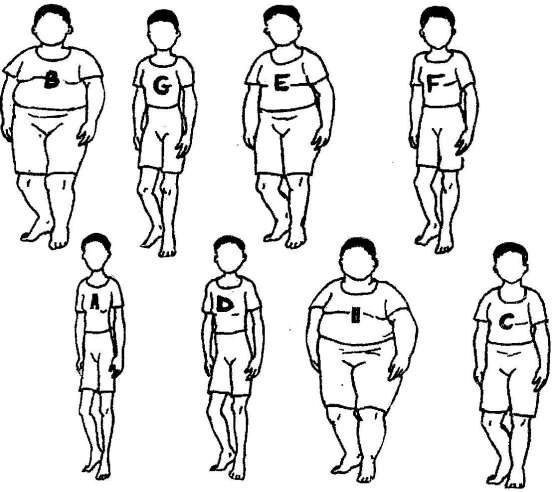


1. Which of these boys do think resembles your current weight?

**THANK YOU**

**WE HAVE REACHED THE END OF OUR QUESTIONS**

**______________________________________________________________________________**
