# Supplementary material for: Level of agreement between objectively determined body composition and perceived body image in 6- to 8-year-old South African children: The Body Composition–Isotope Technique study
Source: PLoS One. 2020 Aug 10;15(8):e0237399. doi: 10.1371/journal.pone.0237399 (PMC7417193; doi:10.1371/journal.pone.0237399)
Supplement: S2 File — (PDF) [file pone.0237399.s003.pdf]

African Journal for Physical Activity and Health Sciences (AJPHES) Volume 25(2), June 2019, pp. 142-173.

Protocol for validating simple measures of body fatness and physical activity of children in twelve African countries: The ROUND-IT Africa Study

A. EL HAMDOUCHI,[1] T. ADOM,[2] A. AOUIDET,[3] V.D. AGUEH,[4] A. DIOUF,[5] N.I. JOONUS,[6] G.H. LEYNA,[7] K.D. MBITHE,[8] T. MOLEAH,[9] M.A. MONYEKI,[10] D. NAKIMBUGWE,[11] H.L. NASHANDI,[12] A.F. SIMPARA,[13] S.M.A. SOMDA,[14] J.J. REILLY[15] AND C. LOECHL[9]

[1]*Unité Mixte de Recherche Nutrition et Alimentation CNESTEN- Université Ibn Tofail (URAC39), Regional Designated Center of Nutrition Associated with AFRA/IAEA, Morocco. E-mails: asmaaelhamdouchi@gmail.com; elhamdouchiasmaa@gmail.com or andries.monyeki@nwu.ac.za*
[2]*Nutrition Research Centre, Radiological and Medical Sciences Research Institute, Ghana Atomic Energy Commission (GAEC), P.O. Box LG80, Accra, Ghana.*
[3]*Association Tunisienne des Sciences de la Nutrition (ATSN), Higher School of Sciences and Technology of Health, University of Tunis El Manar, Tunis, Tunisia.*
[4]*Institut Régional de Santé Publique de Ouidah-Cotonou (IRSP), B.P. 918 01, Cotonou, Benin.*
[5]*Université Cheikh Anta Diop de Dakar, Laboratoire de Nutrition, Département de Biologie Animale, Faculté des Sciences et Techniques, Dakar, Senegal.*
[6]*Biochemistry Department, Central Health Laboratory Candos, Victoria Hospital, Ministry of Health and Quality of Life, Quatre Bornes Candos, Mauritius.*
[7]*Department of Epidemiology & Biostatistics, School of Public Health and Social Sciences (SPHSS), Muhimbili University of Health and Allied Sciences (MUHAS), P.O. Box 65015, Dar es Salaam, United Republic of Tanzania.*
[8]*School of Public Health and Applied Human Sciences, Kenyatta University, P.O. Box 43844-00100, Nairobi, Kenya.*
[9]*International Atomic Energy Agency, Division of Human Health, Vienna International Centre, P.O. Box 100, Wagramer Straße 5, 1400 Vienna, Austria.*
[10]*Physical activity, Sport and Recreation Research Focus area, Faculty of Health Sciences, North-West University (NWU), Private Bag X6001, No. 11 Hoffman Street, Potchefstroom 2520, South Africa.*
[11]*Department of Food Science and Technology, Faculty of Agriculture and Forestry, Makerere University, P.O. Box 7062, Kampala, Uganda.*
[12]*Ministry of Health and Social Services, Private Bag 13198, Harvey Street, Windhoek, Namibia.*
[13]*Laboratoire national de la sante B.P. 232, Bamako, Mali.*
[14]*Centre Muraz, 2054 Avenue Mamadou Konate, 01 BP 390, Bobo-Dioulasso 01, Burkina Faso.*
[15]*School of Psychological Sciences & Health, University of Strathclyde, Glasgow G11XQ, Scotland*

(Received: 20 July 2018; Revision Accepted: 28 May 2019)

Abstract

Body fatness and physical activity (PA) among children should be measured accurately to address the pandemics of obesity and physical inactivity. Limited accurate PA data exist in the African region, and crude proxy measures of body composition (such as body mass index: BMI) and physical activity (such as questionnaires) may have limited most surveys and studies from the region. A stable isotope technique (deuterium dilution) and accelerometry provide 'gold standards' for validating measures of body fatness and PA, respectively. The consortium on "Reducing Obesity Using Nuclear Techniques to Design Interventions in Africa (ROUND-IT)" Study,

therefore aimed to validate the accuracy of, 1) World Health Organisation's (WHO) BMI-for-age as a means of assessing excessive body fatness; 2) the Physical Activity Questionnaire for Children (PAQ-C) as a means of measuring PA, and 3) to describe accelerometer-measured PA in a large multi-country African sample of children 8 – 11 years of age. ROUND-IT Africa is a cross-sectional multi-centre study conducted in 12 countries (Benin, Ghana, Kenya, Mali, Mauritius, Morocco, Namibia, Senegal, Tunisia, Uganda, the United Republic of Tanzania and South Africa). The participants of the study were 6 to 11 years old (n=2050). Data collection began in April 2014 and was completed at the end of 2018. Height, body weight and waist circumference were measured using standard methods. Obesity was defined based on WHO's BMI-for-age criteria. Body fatness was measured using total body water, and excess fatness determined using the criterion-referenced thresholds of >25% (boys) and >30% (girls). PA and sedentary behavior were assessed for 7 days in randomly assigned subsamples by accelerometry. The study was approved by local ethics committees in each participating country. Findings of the research, which will add to the existing body of scientific knowledge on African children, will be disseminated through journal publications, technical reports, conferences, and contacts with relevant non-governmental organisations, particularly the WHO.

Keywords: Children, obesity, overweight, body composition, physical activity.

How to cite this article:
El Hamdouchi, A., Adom, T., Aouidet, A., Agueh, V.D., Diouf, A., Joonus, N.I., Leyna, G.H., Mbithe, K.D., Moleah, T., Monyeki, M.A., Nakimbugwe, D., Nashandi, H.L., Simpara, A.F., Somda, S.M.A., Reilly, J.J. & Loechl, C. (2019). Protocol for validating simple measures of body fatness and physical activity of children in twelve African countries: The ROUND-IT Africa Study. *African Journal for Physical Activity and Health Sciences*, 25(2), 142-173.

Introduction

Africa is currently undergoing physical activity (PA) (Muthuri, Wachira, Onywera & Tremblay, 2014) and nutritional (Vorster, Kruger & Margetts, 2011) transitions, which have triggered a growing problem of obesity and related non-communicable diseases (NCDs) in the region. Indeed, in Africa, NCDs accounted for 28% of morbidity and 35% of mortality in 1990 (Brown, Goldie, Draisma, Harford & Lipscomb, 2006). By 2008, age-standardized NCD mortality rates for all ages were highest in the African Region (males - 844 per 100 000 and females - 724 per 100 000) (Alwan, Maclean, Riley, d'Espaignet, Mathers, Stevens & Bettcher, 2010). According to the World Health Organisation's (WHO) Global Status Report on NCDs in 2010 (WHO, 2014), global NCD related-deaths are projected to increase by 15% between 2010 and 2020, with a 20% anticipated increase of NCD related-deaths in low- to middle-income countries in Africa, the Eastern Mediterranean, and South-East Asia.

Timing of the PA and nutrition transitions has varied across the African region, but in most of the region, there is evidence of increasing obesity and diabetes. In Botswana, for example, the national prevalence of hypertension was estimated to be 16.9% (9.9% for males versus 18.9% for females) (Keetile, Navaneetham & Letamoz, 2015), while in Morocco, 45% of women were already overweight by the early 2000's (Benjelloun, 2002), and 12% of adult men and 18% of adult

women in Tunisia suffered from obesity in 2001 (Kamoun, Hajem, Imen, Achour & Slimane, 2008). This is indicative of the swift changes that the African region is undergoing in its social and economic environments with concomitant changes in food consumption patterns, urbanization and lifestyle. These trends signal an increasingly sedentary lifestyle and over-nutrition, which trigger the growing problem of obesity and related NCDs (Popkin, 1998). Childhood obesity is an emerging problem in the African region, and therefore accurate situation assessments are required to guide the design of intervention strategies to prevent and control it, and to evaluate the impact of these strategies. The World Health Organization considers childhood obesity as one of the most serious public health challenges of the 21st century, despite increased efforts to address it (Gillespie, Haddad, Mannar, Menon & Nisbett, 2013).

In Africa, the estimated prevalence of childhood obesity, defined as an abnormal or excessive fat accumulation that presents a risk to health, increased from 4% in 1990 to 7% in 2011, and is expected to reach 11% in 2025 (Black, Ezzati, Grantham-McGregor, Katz, Martorell, Uauy, Maternal and Child Nutrition Study Group, 2013). In South Africa, the prevalence of overweight among children increased from 10.6% to 18.1% in the age group of 2 – 5 years (Shisana, Parker, Hoosain, Naidoo, Hongoro, Mchiza, Steyn, Dwane, Makoae, Maluleke, Ramlagan, Zungu, Evans, Jacobs, Faber & SANHANES-1 Team, 2013). There is also increasing evidence of declining PA and low cardiorespiratory fitness in children and adolescents in Africa (Muthuri et al., 2014). Recent international comparisons suggest that the levels of PA among African children and adolescents are often very low, though the quality of PA measures in many countries is limited (Tremblay, Liukkonen, Maddison, Ocansey, Onywera, Prista, Reilly, Rodríguez, Martínez, Sarmiento Duenas, Standage & Tomkinson, 2015). The decline in PA has been associated with higher socioeconomic status, urbanization and active transportation in school children (Muthuri et al., 2014). Obesity and inactivity are also found to be associated with low or poor physical fitness as well as elevated blood pressure in children and adolescents (Monyeki, Koppes, Kemper, Monyeki, Toriola, Pienaar & Twisk, 2005; Monyeki, Neetens, Moss & Twisk, 2012; Awotidebe, Monyeki, Moss, Strydom, Amstrong, Kemper, 2016).

Children who are obese are likely to remain obese until adulthood and are more likely to develop NCDs like diabetes and cardiovascular diseases at a younger age (Reilly, Dorosty, Ghomizadeh, Sherriff, Wells & Ness, 2010; Reilly & Kelly, 2010). Childhood obesity thus adds a great burden to the existing under-nutrition problem in children in Africa.

To better understand the distribution and health consequences of obesity, a number of studies have been conducted in different African populations (Armstrong, Lambert, Sharwood & Lambert, 2006; Oulamara, Agli & Frelut, 2009; Guthold, Cowan, Autenrieth, Kann & Riley, 2010; Micklesfield, Pedro, Kahn, Kinsman,

Pettifor, Tollman & Norris, 2014). In 2014, an estimated 41 million children under five years of age were affected by overweight or obesity (UNICEF, WHO, World Bank, 2015), as cited in WHO 2016 report on ending childhood obesity (WHO ECHO Report, 2016). It was revealed that, of the identified children who were overweight, 48% lived in Asia and 25% in Africa (WHO ECHO Report, 2016). Although the increase in childhood obesity is frequently attributed to a decline in PA, a remarkable lack of consistency exists in the relationship between reported levels of PA and degrees of fatness (Parsons, Power, Logan & Summerbell, 1999; Goran, Nagy, Treuth, Trowbridge, Dezenberg, McGloin & Gower, 1997). The inconsistency in published studies is probably due to the use of a variety of assessment methods for estimating body composition and PA thereby limiting comparisons across countries. In most previous studies, methods for assessing body composition or PA in children have relied on subjective and invalid techniques, thus questioning their usefulness in the African region (Craig, Reilly & Bland, 2013).

The use of BMI-for-age is the most widely accepted, simple proxy for body estimating composition in children and adults, as recommended by WHO (de Onis, Onyango, Borghi, Siyam, Nishida & Siekmann, 2007) and supported by a systematic review (Reilly & Kelly, 2010). BMI-for-age has been used to assess obesity in individually in children and adolescents as well as for public health surveillance (NHLBI Obesity Education Initiative, 2000; Luke, Bovet, Forrester, Lambert, Plange-Rhule, Dugas, Durazo-Arvizu, Kroff, Richie & Schoeller, 2013). Obesity is in fact a high level of body fatness while BMI-for-age is a weight-based proxy measure for high body fatness. In western adults and children there is substantial evidence that BMI estimates of obesity are conservative, and that BMI is a poor indicator of percent body fatness (Reilly & Kelly, 2010; Reilly et al., 2010; Shah & Braverman, 2012; Javed, Jumean, Murad, Okorodudu, Kumar & Somers, 2015). In addition, the relationship between BMI and body fatness varies between human populations when BMI is used as a proxy for body fatness. For example, in Asian children and adolescents the extent to which BMI underestimates high body fatness is greater than similar estimates obtained from European populations (Nightingale et al., 2011; Liu, Byrne, Kagawa, Ma, Poh, Ismail, & Hills, 2011). However, the extent to which BMI under- or over-estimates body fatness in African children is unknown (Reilly et al., 2010).

Previous attempts to validate methods for assessing body composition and PA in Africa (Mciza, Goedecke & Lambert, 2007; Luke, Durazo-Arvizu, Rotimi et al., 1997; Prins, Hawkesworth, Wright, Fulford et al., 2008; Oyeyemi, Moss, Monyeki & Kruger, 2016) have been undertaken separately in specific countries thereby limiting the opportunity to clearly interpret such findings from a continental perspective. Therefore, there is a need for validation of body composition protocol against a more robust "gold standard" reference method for measurement of body fatness such as total body water (TBW) (Wells & Fewtrell, 2006), which is

especially valuable as it can be used in field settings in low- and middle-income countries (Liu et al., 2011).

Future public health surveillance studies of PA among African children and adolescents would benefit from the use of a relatively simple questionnaire validated against a more direct measure of PA such as accelerometry. At present, even the most widely used PA questionnaires are generally based on limited validation evidence of this kind (Biddle, Gorely, Pearson & Bull, 2011). The Physical Activity Questionnaire for Children (PAQ-C) appears to be the most promising option but has not been validated against accelerometry in contemporary African populations thus emphasising such need. The PAQ-C is a nine-item, seven-day PA recall instrument designed for use in a field-based setting and has been validated as a self-report measure of PA which is widely used to assess PA in children (8–14 years of age) (Crocker, Bailey, Faulkner, Kowalski & McGrath, 1997).

The aims of the ROUND-IT (Reducing Obesity Using Nuclear Techniques to Design Interventions) Africa Study are therefore, to: 1) validate the accuracy of WHO BMI-for-age as a means of assessing excessive body fatness; 2) validate the (PAQ-C) as a measure of PA; and, 3) describe accelerometer-measured PA in children in a large international African sample. The results of the present project, RAF6042: "*Applying Nuclear Techniques to Design and Evaluate Interventions to Reduce Obesity and Related Health Risks,*" upon which the ROUND-IT Africa Study consortium was based, will provide important new information to facilitate our understanding of the obesity epidemic and PA transition in African countries. Findings of the study will also be useful to develop lifestyle and environmental interventions needed to curtail the growing prevalence of childhood obesity across Africa in the longer term.

Methodology

Study settings

ROUND-IT Africa is a multi-country cross-sectional study conducted in 12 countries (Republic of Benin – a narrow, key-shaped, north-south strip of land in West Africa; Ghana– situated along the Gulf of Guinea, just a few degrees north of the equator; Kenya – located with a coastline on the Indian Ocean, which contains swamps of East African mangroves; Mali– a landlocked nation in West Africa, located southwest of Algeria, extending south-west from the southern Sahara Desert through the Sahel to the Sudanian savanna zone; Mauritius– located about 2,000 kilometres (1,200 miles) off the southeast coast of the African continent; Morocco – spans from an area between the Mediterranean Sea and Atlantic Ocean to the north and west, respectively, with large mountainous areas in its hinterland, to the Sahara desert in the far south; and Namibia – driest country

in sub-Saharan Africa, its western border is the Atlantic Ocean and it depends largely on groundwater. Other countries included Senegal – located on the western coast of the continent; Tunisia – the northernmost country of Africa and the smallest country in the Maghreb region of North Africa; Uganda – located in east Africa, west of Kenya, south of South Sudan, east of the Democratic Republic of the Congo, and north of Rwanda and Tanzania; the United Republic of Tanzania– located in the east coast of Africa and South Africa- situated in the Southern Africa region) (Wikepedia, 2019). The International Atomic Energy Agency (IAEA) provided technical support and funding for the ROUND-IT Africa Study.

Study sample

The recruitment goal of ROUND-IT Africa Study was to enroll at least 150 children aged 6 to 11 years old from each of the 12 study countries (N = 2050), a sample size which is much larger than most previous validation studies of this kind (Reilly et al., 2010). The study did not use a sample size calculation because the intended total number of participants of around 2050 was large enough to restrict uncertainty about the classification accuracy of BMI (Altman, 1991). For methodological studies, which aim to validate BMI or PAQ-C, a representative sample is desirable but not essential, though having a large enough sample size to provide a wide range of BMI, body fatness, and PA is important. In an effort to improve comparisons across countries and to examine obesity and its related behaviors in settings where the PA and nutrition transitions may have already taken place (i.e. urban areas) the sampling frame focused on randomly selected samples of school children from urban areas.

In each country, a multi-stage random sampling technique was used to select at least 4-5 urban public schools in one district or state followed by school sampling frames of all classes corresponding to the target age group and gender (Table 1). On average, the aim was to recruit around 150 children per country; an exception was made to exceed n=155 where desirable. Such exceptions depended on the availability of the participants and the capacity and priorities of each individual country. Children meeting the inclusion criteria were randomly recruited to participate in the study upon submission of a signed parental informed consent form. Additionally, children were requested to sign an assent form during the day of measurements. Data collection was conducted during the school year.

Table 1: Sampling of schools and methods of questionnaire administration by participating country.

Country	Sampling of schools	Questionnaire administration
Ghana	Four schools were randomly selected from a list of public schools obtained from the Municipal Education Directorate. The school heads and teachers were sensitized about the objectives, procedures and outcomes of the study. Children aged between 8-11 years in each class and who met the	This was done by putting children into groups of 4-5 and a research assistant was assigned to each group. The research assistants read out the questions and then supervised the children to complete the learner questionnaires.

	inclusion criteria were eligible. Information sheets and consent forms were given to the children for guardian/parental consent. All children who returned the signed consent forms and gave their assent were enrolled.	
Kenya	The study adopted a cross-sectional design which was conducted among children aged 8–11 years from four randomly selected public primary day schools in Kasarani Sub-county in Nairobi city. The city was purposively selected due to rapid urbanization, transition in dietary practices and lifestyle. Kasarani sub-county was randomly selected. Four primary schools were randomly selected from 17 public primary day schools in the selected sub-county. Proportionate to size sampling was used to sample 202 children. At the school level, stratified random sampling was used to select children from each age group (8 years, 9 years, 10 years and 11 years). A representation ratio of boys to girls as 1:1 was anticipated. Actual sample numbers of boys and girls, who participated in this study were 93 boys and 103 girls.	Pupils were grouped into five. Each group of five was supervised by a research assistant. Questionnaires, pencils, sharpener and eraser were issued to each child. Each child filled his/her own questionnaire. The group of five started together, and with the guidance/explanations/supervision of the research assistant they all completed one question before moving to the next until the questionnaire was completed.
Mali	The schools were chosen randomly in the six communities of Bamako by the focal point of the Ministry of Education, with the agreement of the Centers for Pedagogic Animation. One school in each communal district was selected, in total six schools, out of which three were public and three were private. From the pupil list supplied by the schools, the pupils were numbered and classified by sex. It was planned to select 36 pupils (18 boys and 18 girls) per school. Therefore, a « Random Number Table » of the software Emergency Nutrition Assessment (ENA) was applied in order to obtain the list of selected pupils by sex and by school. When the number of consented pupils/parents was not sufficient, additional pupils were selected from those who volunteered and by sex.	The questionnaires were administered individually to each child by direct interview.
Mauritius	Written official consent was obtained from the Ministry of Education and Human Resource, Tertiary Education and Scientific Research after a meeting with the stakeholders. The Ministry of Education proposed 6 schools in the urban and suburban region of Quatre-Bornes, covering a catchment area known as zone 4 and we recruited	An interview-based administration of the questionnaire was preferred. The questionnaire explored the fact that the participants were all currently in the school system and had recess and school breaks as a regular part of their school week.<br>Trained interviewers helped to translate the questions into simpler terms especially for the very young participants and those who had

	subjects from 4 of them. We had the collaboration of the technical staff and the teachers of these respective establishments to set up a clinic in the school premises for recruiting participants and collecting data amongst the school children.<br>All participants were chosen at random as we selected the whole class to participate in the survey. We targeted participants in the 4th-5th and 6th standards.	some limitations in their linguistic skills. Interviews were carried out during normal school hours just while taking the blood pressure measurements.<br>The school environment survey and audit was also carried out to assess the physical, social and policy environment in terms of healthy eating and physical activity in the schools. We completed the survey by interview with a key informant (principal, teacher, administrative staff, and caretaker) and by observation.
Morocco	Recruitment targeted a sex and age balanced sample of 252 children between the ages of 8 and 11 years. The primary sampling frame was a convenience sample of public schools in Kenitra and Rabat. Schools which accepted to take part in the study were recruited. The secondary sampling frame consisted of classes in the recruited schools that would best yield a final sample with sex and age balance. In each class children were approached to participate in the study and were sent home with an introduction letter and consent form for their parents or guardians to review and give consent. Children were thereafter asked to provide assent to participate in the study.<br>Children who met the inclusion criteria and whose parents signed consent forms were included in the study.	Administration of lifestyle questionnaire was done to groups of 1-2 children assisted by one researcher of the team.
Namibia	There were four schools involved in this study whereby a school was randomly chosen from different educational zones of Windhoek, Khomas region. At school level, children who met the inclusion criteria were randomly selected from different classes and only those whose parents signed consent forms were included in the study.	A total of 12 data collectors were involved in the study. A team worked from one school to another. The team was given a room in the school where they operated and set up their measurement points. Each child was interviewed by the interviewer using the learner questionnaire and the child responded to the questions being asked. Each child went through all the points until all the procedures were done as planned. Children were given the lunch pack after the last saliva sample was taken.
Senegal	The study was a cross sectional design conducted in December 2013 among 8-11 year old school children in urban areas of Dakar. A two-stage stratified sampling was used. In the first stage, four elementary public schools were randomly selected from the 149 of the Academic Inspection of Dakar. In the second stage, 156 pupils (76 boys and 80 girls) were randomly recruited from separate lists of girls and boys in each school. The study was the “Comité	A structured questionnaire was used to collect socio-economic status of the learner, nutrition knowledge, attitude and practice, sedentary lifestyles and physical activity using face to face interviews. For standardization purposes, the questionnaire was first tested among 10 children in one elementary school. This approach has subsequently allowed to harmonize the administration of the questionnaire and also to estimate the duration of the interview. The questionnaires were

	d'Ethique de la Recherche de l'UCAD (0011/2014/CER/UCAD) and authorized by the Ministry of Education (MoE). Meetings with the MoE through the Inspection Academy of Dakar took place. Before starting the study, several meetings were organized with de Division of Medical Control School (principal partner). After that, two awareness meetings were conducted in each school. One with the pedagogical team and the association of parent pupils, and a second one with the learners and their parents. The aims of theses meetings were to inform about the study objectives and procedures as well as the selection criteria of the children. Informed parental consent and child assent were obtained from all participants before enrolment. All the data were collected during the full school year.	reviewed at the end of each interview to correct any mistakes or missing questions.
Tunisia	Before beginning the study, we obtained the approval of the ethics committee of the National Institute of Nutrition and Food Technology and the agreement of the Ministry of Education. A total of 156 children (80 boys and 76 girls) aged between 8 and 11 years were recruited from three randomly selected primary schools. The sample selection was conducted randomly at two levels: schools and children. The primary sampling concerned the public schools in Manouba. Schools whose directors accepted to take part in the study were recruited. The secondary sampling concerned children. Children with a good general health and whose parents signed consent forms were included in the study.	The questionnaires were administrated individually to each child by a direct interview to collect information related to dietary patterns, lifestyle and physical activity. The interview-based questionnaire was administered by the research assistants.
Uganda	The sample was recruited using a multistage method. In the first stage, a registry of public schools was obtained from each of the five divisions from KCCA directorate of Education and Social Services. A convenient list of public schools within a division was written on a sheet. A school was approached to be part of the study depending on proximity, time, accessibility and cost. When the school objected it was replaced by a willing randomly selected school. In the second stage, classes 3-6 were selected for the study. Children's ages were availed by class teachers from the class registry and those that were within the	The questionnaires were self-administered by the children, with support from teachers and researchers. The researchers explained the questionnaire components and procedures for completing it to both the children and teachers in an introductory session. Teachers divided the selected children into groups of 5-10. The teachers repeated the instructions earlier explained by the researchers. The teachers then read out each question to the children, as the children also read from their own questionnaires and answered the questions. Children were free to ask questions to the teachers as necessary. The teachers could also consult the researchers for clarifications when necessary.

	age bracket of 8-11 yrs were included for recruitment. Twenty-four (24) girls and 24 boys were randomly recruited for enrolment into study. The names of girls and boys in the class were written down in an alphabetical order on different sheets of paper by the teacher. The pupils whose names were in odd numbers in the names lists for boys and girls were included in the study. Six (6) boys and 6 girls were recruited in the study by picking one from the most top, middle and below alternately to get 6 boys and girls. Twelve (12) pupils were recruited from each class and 48 from each school. Pupils were given copies of the information sheet including informed consent forms, seeking for voluntary participation of children in the study. A direct contact phone number of the researcher was included to take home to their parents. School officials indicated that they routinely send information home to parents in this way. Parents were instructed to contact the researcher directly if they did not want their child to participate in the study. Children whose parents returned signed informed consent were enrolled into the study.	
United Republic of Tanzania	A multi-stage random sampling was used to select schools and recruit study participants. One district of the Dar es Salaam city was randomly selected followed by one (1) ward. Five (5) schools were then selected from the ward and 30 pupils from each school. Pupil selection was stratified by age and sex. A total of 150 children aged 8-11 years were recruited.	A self-administered questionnaire was used to collect information on demographic characteristics, knowledge, attitude and behaviors related to nutrition and dietary patterns, physical activity and sedentary lifestyle. The data collection process mimicked an examination where students sat apart to minimize sharing of information. The investigator walked the study participants through the questionnaire, one question at a time and elaborations were made where necessary.
Benin Republic	The schools and schoolchildren were randomly selected. The school children in the study sample attended schools all of which were located in Cotonou and belonged to the four school districts in the urban and peri-urban areas of the city. A public school was chosen by simple random draw in each of the four school districts. In each of these four schools, the choice of children was made in the classes of CE1 CE2 and CM1 by a systematic random draw from the list of schoolchildren aged 8 to 11 years. A total of 182 schoolchildren were surveyed. The number of schoolchildren selected by	The data were collected in schools by a team of four collection officers, four supervisors and three coordinators, all of whom were trained beforehand to complete the questionnaires. The composition of the team was as follows: two teacher researchers from the Ouidah Regional Public Health Institute (Benin) and an agro-nutritionist to ensure coordination; two medical students and two nutritionists to provide supervision; four graduate students in nutrition and dietetics to ensure data collection; one senior laboratory technician to collect saliva samples. Data collection took place over five days in the four schools. In each selected class, questionnaires were administered to schoolchildren by

	school was based on the weight of the school enrollment among the four deductions.	interviewers who explained difficult questions to them to facilitate understanding and limit bias in their responses. At the end of the questionnaire interview, the weight, height, waist circumference and blood pressure of the pupils were measured and reported on the cards after checking, according to the procedures used for the study. The saliva samples were then taken. At the end a snack was offered to each schoolchild.
South Africa	Five primary schools in urban and township areas were randomly selected in the Tlokwe Local municipality of the North West Province of South Africa with the help of the district Department of Education. The schools were purposively selected due to the rapid nutritional dietary practice and life style transitions. Children aged 6 to 8 years were randomly selected to participate in the study. School register was used to select the children within the desired age limits, of which every third child within the age category was afforded an opportunity to participate in the study. It is expected that the study will recruit a total of 200 boys and 200 girls at the end (June 2018) of the measurement period. The Education Department granted the permission for the study in 27 June 2017. Health Research Ethics Committee of the Faculty of Health Sciences (HREC)) of the North-West University approved (Ethic no: NWU-00025-17-S1) the study.	The questionnaires were administered individually to each child by a direct interview conducted by trained research assistants.

Questionnaires were administered individually in most cases, or by groups of 1-10 children, supervised by a research assistant or mimicking a class examination session (for additional information, see Table 2), followed by measurement of height, weight and waist circumference. Pre- and post-dose saliva samples (enriched with deuterium oxide – D_2O) were collected to assess body fatness using the total body water (TBW) method.

PA was measured by a questionnaire using the PAQ-C. In a subsample of children (~50 children per country, total sample of 550), PA was measured objectively using an Actigraph accelerometer (Model GT3X-BT, Fort Walton Beach, FL, USA). An environmental scan using a pre-designed checklist and interviews with school management were also undertaken (see Table 2).

Table 2: Overview of data collected in the ROUND-IT study.

Variables	Indicators	Tools
Anthropometry (weight, height)	BMI Z-Scores (Plasqui, Bonomi & Westerterp, 2013)<br>Overweight: >+1SD<br>Obesity: >+2SD	Recording form<br>Infant/Child/Adult Portable Height-Length Measuring ShorrBoard<br>SECA 869 scale
Waist circumference	Waist-height ratio (Mokha, Srinivasan, Dasmahapatra, Fernandez, Chen, Xu & Berenson, 2010) - ≥ 0.5 centrally obese	The Lufkin tape measure
Body composition – TBW	% body fat (based on TBW)<br>>25% excess body fat in boys: over fatness<br>>30% excess body fat in girls: over fatness	Recording form<br>FTIR
Bioelectrical Impedance	impedance and body fat	Various devices used; impedance data were not part of the core ROUND-IT study and are reported separately
Physical activity – standardized questionnaire	Score of physical activity from 1-5 (Rowlands & Eston, 2007).	PAQ-C questionnaire
Accelerometry	Minutes of moderate-to-vigorous physical activity (MVPA), sedentary time, (Evenson and Puyau cut points (World Health Organization, 2008; Reilly, 2010).	ActiGraph GT3X+, wGT3X-BT
Blood pressure	Systolic hypertension was defined as systolic BP (SBP) > 95th percentile (equal to a Z-score of 1.64) for gender, age and height.	Recording form<br>Omrom digital device
Demographics and socio-economic status	Socioeconomic status of family, residential address, health history of participant and parents, family structure, age, education	Standardized Questionnaire
School environment	School facilities, healthy eating and physical activity policies, extracurricular activities, frequency of physical education, availability of healthy and unhealthy food in proximity of schools.	Standardized Questionnaire

Lifestyle, demographic, environmental, food and nutrition data

Data on demographic characteristics, including age and gender; measures of socioeconomic status (SES) such as the number of rooms in the children's household, the number of persons living in the household, asset ownership, the main source of drinking water, and sanitation facilities, were collected. Other information obtained included household food insecurity, perceived body image, knowledge of healthy eating and other eating behaviors. The questionnaires took about 30 minutes to complete on average.

Energy balance related behaviors were measured using the questionnaire provided by the South Africa group (Division Exercise Science and Sports Medicine,

Department of Human Biology, Faculty of Health Sciences, University of Cape Town), and adapted for the purpose of this study. This questionnaire was based on a compilation of instruments obtained from the Physical Activity Neighborhood Environment Survey (PANES), School Health Environment Survey SHAPES project, Situational and Observational analysis of the Health Kick World Diabetes Foundation Project in South Africa: the Alliance for a Healthier Generation Healthy School Inventory and Centers for Disease Control School Health Index, as well as the School Healthy Policies and Practices Survey. All ROUND-IT Study questionnaires and resources are obtainable from the corresponding author upon request.

An individual child questionnaire was completed by children in the presence of research team member and school teacher. The questionnaire included items related to food consumption, PA, sedentary behavior, health and well-being, and was administered to all participating children. The interviewers were trained to administer the questionnaire in a standardized fashion in order to minimise bias. Questionnaires were checked for completeness at the time of data collection in order to ensure, as described above, quality of the data collected and data management procedures.

Physical activity measurement by questionnaire

In many countries, PA is still measured by questionnaire for research and surveillance purposes. Of the many questionnaires available, the PAQ-C has high practical utility, has been used widely, and has some evidence of validity relative to objective measures from high-income countries (Kowalski et al., 2004 Wang, Baranowski, Lau, Chen & Pitkethly, 2016).

The PAQ-C was originally designed for 8 to 14-year-old children and makes reference to a student's last 7 days' PA recall, activities during PE, lunch break, after school, in the evenings and weekends. It produces a total score ranging from 1 to 5, obtained from 9 PA domains, of which each has 5 parts. Finally, the mean of the items is used to calculate the final PAQ-C summary score. The summary score is then classified using Kowalski et al. (2004) scores: Light (score = 1), Moderate (score = 2–4), and Vigorous (score = 5) that indicates a higher level of activity. In this study, only children aged 8 to 11 years completed the questionnaire.

School environmental questionnaire

The school environment was assessed by a combination of a questionnaire completed by a school administrator (e.g. school director) and a direct audit of the school environment, performed by a ROUND-IT Africa Study researcher. The school environmental questionnaire was divided into 4 sections: School demographics and neighborhood environment (socioeconomic status of the learners within the school and how learners travel to school, the community's

surroundings and the physical environment of the neighborhood around the school); School health, PA and nutrition environment (condition of the school/buildings/surroundings, the amenities/facilities which are available at the school, the number of playgrounds/sport fields for learners and their conditions); School feeding programme/nutrition policies/vendors (which contains questions such as: Is there a tuck shop/snack shop/canteen at the school? Is there a vegetable garden at the school? If so, please indicate the purpose of the garden and the food produced. Are there formal or informal vendors at or adjacent to the school?); and Health challenges for learners and teachers (consisting of: tobacco use, substance abuse, lack of PA, unhealthy diet, overweight, underweight, chronic diseases of lifestyle, e.g. diabetes, heart disease, high blood pressure, health problems related to issues of sexuality, e.g. HIV/AIDS, teenage pregnancy) (Reddy, James, Sewpaul, Koopman, Funani, Sifunda, Masuka, Kambaran & Omardien, 2010; de Villiers, Steyn, Draper, Fourie, Barkhuizen, Lombard, Dalais, Abrahams & Lambert, 2012).

Measurements

Anthropometry

Anthropometric measurements were taken according to standardized procedures established at the project-training meeting in September 2012. Weight was measured to the nearest 0.1 kg using an electronic portable scale (SECA model 869, Germany), with the participant barefoot and wearing light clothes. Height was measured to the nearest 0.1 cm using a stadiometer (ShorrBoard, Portable Height-Length Measuring Board) with the participant barefoot in a standing position and all hair accessories removed from the head. Two readings were recorded. Body mass index (BMI) was calculated as the weight (kg) divided by the square of the height (m). Overweight and obesity were classified according to WHO's BMI cut-offs for children (de Onis et al., 2007). Z-scores were calculated using WHO Child Growth Charts and WHO 2007 Reference Charts (WHO, 2007). The cut-off values for BMI-for-age used were overweight: >+1SD and obesity: >+2SD.

Waist circumference was measured at the end of normal expiration with a non-elastic tape held midway between the lower rib margin and the iliac crest (WHO, 2011). Waist circumference is a reasonable proxy for total body fat and cardio metabolic risk in children, according to studies from high-income countries (Reilly et al., 2011). A cut-off waist: height ratio of >0.5 has been proposed as a simple universal indicator of excess total body fatness and/or excess central body fatness in both adults (Li, Chen, Chang, Loke, Wang & Hsiao, 2013) and children (Kuba, Leone & Damiani, 2013).

Blood pressure

There is substantial evidence that blood pressure (BP) is raised by obesity, even in children and adolescents (Hanevold, Waller, Daniels, Portman & Sorof, 2004; Raj & Krishnakumar, 2013), but limited BP data on African children and adolescents BP are available. For the present study, BP was measured with the oscillometric device Omron 705IT (Omron Healthcare Europe), which has been validated for use in children and adolescents (Stergiou, Yiannes & Rarra, 2006). Appropriate cuff sizes were used in conformity with the manufacturer's recommendation: small (AC < 23 cm), medium ($23 \leq$ AC < 32 cm), and large (AC ≥ 32 cm). With the appropriate cuff size, individuals had three BP measurements made after a minimum of ten minutes of rest in the supine position in a quiet and well ventillated room. The mean of the last two of three BP measurements made was reported and Z-scores calculated according to an American reference standard based on gender, age and height (*National High Blood Pressure Education Program Working Group on high blood pressure in children and adolescents*, 2004). Systolic hypertension was defined as systolic BP (SBP) > 95th percentile (equal to a z score of 1.64) for gender, age and height.

Physical activity measurement by accelerometry

It is now widely recognized that objective measurement of PA is important when estimating levels/intensities of PA, and the most widely used device for obtaining such objective measures is the Actigraph (Dinesh & Patty, 2012) accelerometer (ActiGraph, Pensacola, FL). The Actigraph accelerometer was used in the present study to objectively assess PA levels and sedentary behavior in a subsample of children. As with the other measurements in the ROUND-IT Africa Study, accelerometry data were collected and prepared for analysis according to a standard operating procedure. Accelerometry data collection and preparation procedures were similar to those described widely in the literature (Sasaki, John & Freedson, 2011). The accelerometer was worn at the waist level on an elasticated belt, specifically on the right mid-axillary line. Children were encouraged to wear the accelerometer on waking days only for 7 days, including 3 weekdays and 1 weekend day. Children were also requested to give back the ActiGraph accelerometer at their respective schools after completion of the stipulated days of wear. Data were collected in 80 Hz intervals. Raw accelerometer data were downloaded, verified by the research team using the ActiLife software (ActiGraph, GT3XE-Plus, Pensacola, FL, version 6.13.3) available at the time of the study.

Accelerometer data were then summed into 15s epochs. Non-wear time was defined as at least 60 min of consecutive zero counts with a 2 min interruption tolerance (Rich, Griffiths & Dezateux, 2013). The minimal amount of accelerometer data that was considered acceptable was 4 days with at least 10 hours of wear time per day, including at least one weekend day (Troiano et al., 2008; Reilly, 2010; Heil, Brage & Rothney, 2012). Additional support (expert

visits and trainings) was provided to study sites in each country when necessary in order to maintain quality control of accelerometer data collected.

Physical activity measurement by step counting
Step counting is a simple and widely accepted objective measure of PA, and levels of step counts per day for children and adolescents which correspond to the recommended levels of moderate to vigorous physical activities (MVPA) (Tudor-Locke, Craig, Beets, Belton, Cardon & Duncan, 2011) are being increasingly used. In the ROUND-IT Africa Study, daily step counts were estimated by within-instrument processing of the number of cycles in the accelerometer signal or "cycle counts" (Tudor-Locke et al., 2011). The ActiGraph step feature has been validated against directly observed steps with normal-weight and over-weight youth (Arvidsson, Fitch, Hudes, Tudor-Locke & Fleming, 2011; Hallal, Reichert, Clark, Cordeira, Menezes, Eaton, Ekelund & Wells, 2013). Accelerometer step and intensity outputs could potentially inform a pedometer-scaled steps/day value congruent with public health MVPA guidelines. However, relatively few data exist to inform a steps/day translation of the ≥60 minute/day MVPA guideline in children, especially in Africa.

Deuterium Oxide dilution method for measurement of Total Body Water (TBW) and total body fat
As noted above, TBW measurement is a reference method for the measurement of body fatness in children and adolescents (Wells & Fewtrell, 2006) with high accuracy. TBW was assessed by the deuterium dilution technique - a standard method described in detail elsewhere (IAEA, 2010). Children first provided a pre-deuterium dose saliva sample to determine the natural 'background' deuterium enrichment of saliva. Saliva (≈5 mL) was collected directly by moving a cotton wool ball in a closed mouth of the participant until it is soaked. Then, each participating child transferred the cotton wool directly into the body of the syringe. With a plunge pressed into the syringe, the saliva was collected into sterile and labeled vials. Secondly, an oral dose of 0.5 g/kg body weight of a 99.8% deuterium oxide solution was carefully pre-weighed using a scale with a sensitivity of 0.0001 g (Ohaus Explorer Pro EP214 balance) under sterile conditions and was given orally through a straw, to avoid spillage, to the participant.

The bottle containing the dose was rinsed twice with 50 ml of drinking water. The post-dose samples were collected 3h-4h after the administration of deuterium-labelled water to allow time for equilibration of deuterium in body water. All tubes had screw-on caps and were individually placed in zip-lock bags preventing possible losses due to evaporation during tube manipulation and cross-contamination when the sample is being stored. During the equilibration period, children remained in the study location (i.e. classroom or laboratory depending on each member state's logistical arrangements) and were allowed only a small meal as recommended (Schoeller, Van Santen, Paterson, Dietz, Jaspan & Klein, 1980).

The saliva samples were stored at -20°C until analysis by a Fourier transform infrared spectrophotometer (FTIR, Shimadzu 8400S, Kyoto, Japan for nine countries, and by Portable FTIR Agilent 4500, Waldbronn, Germany for the remaining three). The FTIR apparatus was equipped with a calcium fluoride cell of 150 ml volume and 0.1mm path length. A standard solution of 1000mg D_2O per kg of local tap water was gravimetrically prepared and used to calibrate the FTIR. The standard was analyzed at the beginning, in the middle and at the end of every series of measurements. The enrichment of the saliva samples with D_2O were obtained against the standard. Saliva samples were completely thawed at room temperature and centrifuged to flocculate solid particles. The paired pre- and post-dose samples were analyzed in duplicate. The spectra obtained from the FTIR were converted into a deuterium enrichment of saliva (mg/kg) using the Isotope software (Medical Research Council, MRC, Cambridge, UK). The following calculations were performed:

Deuterium space= amount of deuterium consumed/enrichment
TBW_{D2O}= deuterium space/non-aqueous exchange constant (1.041) (Lohman, 1986)
TBW was used to estimate FFM according to age and hydration factor provided by Lohman (Lohman, 1986; Lohman, 1992) for both boys and girls.
FFM_{D2O}= TBW_{D2O}/hydration factor
FM_{D2O} (fat mass) = Body weight—FFM_{D2O}.

The body fatness measures provided by the TBW method were subjected to stringent quality control checks. Individual data points were subjected to data management procedures described below, but in addition each individual post-dose saliva sample, and TBW and body fatness measure were checked. The dataset from each of the participating countries was checked against normal ranges of total body water, % fat-free mass and % fat mass. Quality control procedures for the TBW and total body fat measures are described in more detail in Table 3. The cut-off values for excessive % fat used in the ROUND-IT Study are 25% for males and 30% for females, on the grounds that at those levels of fatness there are substantial increases in cardio-metabolic risk factors in boys and girls, respectively (Williams, Going, Scott, Lohman, Harsha, Snnivasan, Lany, Webber & Berenson, 1992).

Data collection began in April 2014, and was completed by the end of September 2018. Researchers from all 12 countries were trained to use standardized methodology following standard operating procedures (SOPs) for all measures, and quality control checks were performed. The 12 study sites generally used the same measurement protocol, with the exception of the FTIR, as discussed above.

Data management and quality control

In order to harmonize data management across the 12 regions of Africa, a quality assurance and quality control (QA-QC) plan was designed by a biostatistician /data management specialist to facilitate data entry and processing (See Table 3). A single data collection form was made available to all the participating countries in English. Countries were free to add specific questions based on their local needs and preferred language. A standardized data entry form was developed using EpiData software (Lauritsen & Bruus, 2008). Following collection, the data were coded and entered into Epidata (Version 3.1). The form had several data entry controls imbedded in order to avoid errors. In the case of discrepancies, the data in question were confirmed by reviewing the original questionnaires. Double data entry was performed and a data validation programme used. Data cleaning programmes were prepared for each country and for the pooled data. A manual of procedures was used by all centers to support data management and quality control. The manual detailed the software installation procedure, how to enter data from the different questionnaires, how to perform and validate double data entry. The integrity of the data was safeguarded by using a password, which was provided to a biostatistician contracted by IAEA to the project for quality control. In each member state, soft copies of data are kept in a password-protected computer and hard copies of data are securely stored with strict access control.

Data pooling

Plans for data pooling between the 12 participating study sites were agreed by the co-authors at the first co-ordination meeting held in Morocco in May 2012 in whichthe validation of BMI and PAQ-C, using pooled data collection and analyses, were planned. Pooled data were submitted to the offical biostatistician of the IAEA RAF6042 project. Subsequently, the biostatistician organized the data for analysis based on the objectives of the study.

Table 3: Quality control procedures for total body water (TBW) and total body fat measures.

**Basic Quality Control (QC) steps for TBW and % body fat (BF)**
• Screen measurements of deuterium enrichment variation for two post dose samples.
• Calculate expected enrichment and compare with measured enrichment.
• Identify outliers in body composition data using the relationship of TBW and Height3 as QC check.
• Compare calculated %BF with weight, age, height, BMI, sex and WHO standards for weight for age or BMI for age.
• Output range check for total body water (TBW), fat free mass (FFM), fat mass (FM).

Based on the outline in Table 3, the specific details regarding quality control procedures are provided as follows: The first step for checking the quality of data collected is to screen the measurement or assay variation in FTIR readings for the two post-dose saliva samples. They should have values that vary within 2% of their mean. The next step is to calculate the expected enrichment based on the administered dose (mg) over the predicted TBW in kg (7.4 x Height (m)^3) and

compare it to the measured enrichment. The relationship of TBW and Height^3 (m^3) should be used to identify outliers (using <5.7 X height^3 as lower limit and>9.6 X height3 at 95% confidence interval as upper limit). For TBW results outside this QC range, samples should be reanalysed to ascertain the result, or if not possible, they should be excluded. Another quality control step is to compare the calculated %BF with weight, age, height, BMI, sex and WHO standards for weight for age or BMI for age. Data with negative body fat should be excluded, because they may indicate problems with dose consumption, sampling or deuterium analysis. Generally, based on observation, TBW data should be within 37-66% of body weight. Based on body fat reference data for children, FM results should fall between the 2nd and 98th percentile for population specific curves; values outside of these ranges should be investigated further to ensure true result and not measurement error. As a general guide, %FM between 5-58% would be expected for the paediatric population (Schoeller et al., 1980). Based on the above QC steps, results for %FM and %FFM were recalculated using the second post-dose sample reading if the FTIR reading variation was outside the range, or excluded if %FM was negative, less than 5% of body weight or outside the QC range. Only results for TBW with BMI for age at more than +2SD or less than –2SD which were outside the QC range, were accepted.

Training of personnel and quality control
The standardisation of measurement protocols across countries was critical to ensure consistent quality and thus the success of this multi-country study. Regional training sessions were organized by the IAEA for all key staff (See Table 4). Specifically, a workshop on harmonizing project measurement methodologies was conducted in South Africa in November 2012: SOPs on anthropometric, BP and PA measurements were developed and practical sessions carried out over a one-week period. The SOPs for anthropometry were taken from the WHO reference methods and those for BP were extracted from existing protocols (National High Blood Pressure Education Program, 1996; WHO, 2011). In addition, SOPs were used for the study's questionnaires. All countries used the same standard questionnaires as published with little adaptation, which were translated into local languages, using back translation techniques. One exception to this was the PAQ-C questionnaire for the assessment of PA, which was modified to include some country-specific physical activities as necessary (modifications were approved by the lead authors from the 12 countries).

A practical training workshop was conducted in Morocco in May 2013 on TBW measurement and quality control. This training course was run in both English and French, and was provided by the "Unité Mixte de Recherche en Nutrition et Alimentation, Regional Designated Center of Nutrition Associated with AFRA/IAEA" before data collection commenced. An additional regional Training Course on Data Evaluation and Analysis and Quality Control for all the variables being measured in the ROUND-IT Africa Study was conducted in Benin Republic

in November 2014. Project researchers were trained to standardize the information collection process in order to maximize reliability of the data and minimize bias.

Capacity building

As stated earlier, the ROUND-IT Africa Study (RAF6042) was a regional technical co-operation project funded by the IAEA, intended to enhance the research capacity of investigators, technicians, and post-graduate students across the 12 African countries in the measurement of body weight status, body composition, PA and energy balance. The IAEA had no influence on the direction of the research findings. This study was aimed at improving the capacity of researchers from participating country to design studies, to use stable isotope and alternative techniques, and to foster cross-country collaborations. Details of the capacity development meetings and training workshops undertaken are provided in Table 4. The overall long-term aim of the project was to provide valuable information that will help participating countries to understand PA and nutrition transition, and design specific evidence-based interventions.

Table 4: Coordination meetings and training sessions with their objectives under the ROUND-IT study.

First Coordination Meeting, May 2012, Rabat, Morocco
Objectives of the meeting:

- Introduce participants to the procedures for implementation of IAEA-supported projects;
- Promote the concept of ownership of IAEA projects and emphasize the principle of shared responsibility and accountability;
- Identify project objectives related to obesity and finalise the work plan for the project (incl. identify hosts for RTC/meetings; plans for data pooling);
- Identify the Agency's required inputs in support of the project.

Consultative meeting and workshop on harmonizing project implementation and methodologies, November 2012, Cape Town, South Africa
Objectives of the meeting:

- Elaborate SOPs on anthropometric, blood pressure and physical activity measurements.
- Practical sessions.
- Extract SOPs for anthropometry from the WHO reference methods.
- Extract SOPs for blood pressure from existing protocols.
- Modify the PAQ-C questionnaire to include some country-specific physical activities as necessary.
- Modify the learner questionnaire and adapt it to the purpose of the study.

Second Coordination Meeting, September 2014, Vienna, Austria
Objectives of the meeting:

- Review the current situation: progress made and difficulties encountered (country presentations);
- Share initial lessons learnt;

- Review the work plan of the project and action plans for each country project team;
- Review the budget.

Regional Training Course on Data Evaluation and Analysis, November 2014, Cotonou Benin

Objectives of the meeting:

- Provide training in evaluation and analysis of data on body composition assessed by stable isotope techniques, in statistical analysis of national data collected in the studies under RAF/6/042, and in handling of pooled data.
- Revise data cleaning, data quality assurance and data quality control concepts;
- Harmonize statistical analysis (descriptive analysis for important study parameters, inferential statistical methods, types of sub analysis, level of significance) and prepare a statistical analysis plan;
- Analyse data collected in the studies under RAF/6/042;
- Prepare a plan for pooling data from the different studies.

Final Project Coordination Meeting, October 2016, Quatre Bornes, Mauritius

Objectives of the meeting:

- Conduct thorough and in-depth review of the project achievements (planned and implemented activities), share challenges and discuss any remaining activities by each participating country.
- Share national results and lessons learned.
- Pool the data from the participating countries with completed datasets and discuss the type of analysis to be conducted.
- Plan the manuscripts based on pooled analyses.
- Discuss networking beyond the timeframe of the project on interventions related to childhood obesity and related health risks.

Ethical considerations

The ROUND-IT Africa Study protocol was approved by the ethical review committee/boards in each participating country and informed written consent was provided by the parents/guardians of participants (See Table 5). Informed parental consent forms, assent forms and questionnaires were translated into the local language of each study site if necessary. Study leaders/principal investigators at each member states assigned a confidential code to each child known only by the researchers to ensure strict confidentiality and anonymity by use of the confidential code assigned. In the final dataset, it would not be possible to identify specific participants. Participants were informed that drinking stable isotope deuterium water is safe and is associated with minimal risk, because each child was provided with age-specific doses. Fasting is required for determination of TBW by stable isotope, and this may make some of the children uncomfortable. To minimize their discomfort, a light snack and drink were given immediately after the testing procedure. Placing the ActiGraph around the waist may also be uncomfortable at first, but the children were shown how to adjust the belt for comfort. No known risks are associated with wearing the ActiGraph. The role of the IAEA was to assess the overall progress as well as to assist and guide the study.

Support on project research questions, methods, and data analyses were provided by IAEA consultants specializing in the necessary aspects (data management and quality control; data analysis; methodology for measurement of deuterium oxide enrichment in saliva and calculation of body composition, body weight status, and PA by accelerometry).

Table 5: Ethics approval and consent to participate in the study

The ROUND-IT Africa Study protocol was approved by the ethical review committee/boards in each participating country:

- National Ethics Committee (Ministry of Health and Quality of life) in Mauritius;
- Research ethics regulatory committee in South Africa; Health Research Ethics Committee of the Faculty of Health Sciences (HREC) of the North-West University (Ethic no: NWU-00025-17-S1);
- Kenyatta University Ethics Review committee in Kenya;
- Senate Research and publications committee in Tanzania;
- Ghana Health Service Ethics Review Committee in Ghana;
- ‘Comité d'éthique de l’Institut National de Nutrition et de Technologie Alimentaire de Tunisie’ in Tunisia;
- Comité d’éthique et de recherche biomédicale (CERB) in Morocco;
- Research Unit at Ministry of Health and Social Services in Namibia;
- Higher degrees research and ethics committee in Uganda;
- Comité d’éthique de l’Institut national de recherche en santé publique (INRSP) in Mali;
- Comité d'éthique de la Recherche (CER) de l'Université Cheikh Anta Diop de Dakar in Senegal.
- Comité d’Ethique de la Recherche de L’Institut des Sciences Biomédicales Appliquées (CER-ISBA), Ministère de l’Enseignement supérieur et de la Recherche scientifique, Bénin

Statistical analysis-data pooling to address the primary research questions

The ROUND-IT Africa Study was used to answer a range of research questions within each participating country, with multiple additional studies and postgraduate research linked to it. For clarity, considerations for the statistical analyses were restricted to the two main research questions which were answered by data-pooling across the participating study sites: 1) what is the validity of WHO BMI-for age for the assessment of excess body fatness as measured by the reference method based on TBW; and 2) what is the validity of the PAQ-C for the assessment of PA as measured by the objective reference method (Actigraph accelerometry).

In the case of the validation of WHO BMI-for age, the analysis was based on assessing the ‘diagnostic accuracy’ (e.g. sensitivity, specificity, predictive values (Reilly, 2006) of the WHO definition of obesity (BMI Z-score >2.0) (WHO, 2008), and further, receiver operating characteristic curve (ROC) analyses were performed to examine the accuracy of alternative definitions of obesity based on BMI. In the ROUND-IT Study, we used the definition of excess body fatness provided by the seminal study of Williams (Williams, 1992) in which body fatness

measured by a highly accurate multi-component method was related to the criterion of cardio-metabolic risk as described above. Based on previous systematic reviews of the diagnostic accuracy of BMI for age in non-African populations (de Onis et al., 2007; Javed et al., 2015), it was felt that a sample size of around 100-150 children per study site would provide a sufficiently large sample as described above. For the PAQ-C validation, the questionnaire provides scores from one (1) to five (5) (i.e. higher values representing higher levels of PA) and the Actigraph accelerometry data provides continuous and categorical data in different units (e.g. the accelerometer count per epoch is a widely accepted measure of the total volume of PA (Crocker et al., 1997; Kowalski, 2004); therefore, the validation considered the relative validity of the PAQ-C: to determine the extent to which the PAQ-C correctly classifies the most and least active participants as measured by the Actigraph accelerometry method. The PAQ-C has been shown to be internally consistent with Cronbach α between 0.70 and 0.74 in children aged 8–14 years (Moore, Hanes, Barbeau, Gutin, Trevino & Yin, 2007). Based on previous validation studies of PA questionnaires in non-African populations of children and adolescents (Benıtez-Porres, Lopez-Fernandez, Raya, Alvarez, Carnero, Alvero-Cruz & Alvarez Carnero, 2016), it was felt that a pooled sample size of around 30-40 randomly selected participants per study site would provide an adequate sample size. Cross-national data collected are being analysed and will be pubished in future reports.

Discussion

The primary aims of the ROUND-IT Africa Study were (1) to establish the accuracy of standard, widely used and recommended, simple proxies of obesity (WHO BMI definition), and (2) to evaluate the accuracy of the simple and widely used measure of PA (PAQ-C). The study's secondary aims were: (1) to build research capacity in the measurement of body weight status, body composition, and energy balance behaviors across a range of African countries; (2) to begin to collect data on obesity and energy balance related behaviors which would form the basis of a situation assessment in each country; (3) to focus on urban areas where the PA and nutrition transition has had the greatest impact on childhood obesity in Africa to date; (4) to form a consortium of research collaboration in this area across and beyond Africa; and lastly (5), to answer specific relevant research questions linked to the ROUND-IT Africa Study for each participating country.

In time, it is envisaged that the ROUND-IT Africa Study would improve the capacity of African countries to respond to the PA and nutrition transition phenomena in childhood and early adolescence that are periods highlighted as being important to the WHO's Ending Childhood Obesity Commission (WHO, 2016).

The strengths of the ROUND-IT Africa Study include: the extent of African inclusion, from North-South and East-West, with 12 participating countries; the availability of a reference method of objectively measuring PA by accelerometry and body composition by TBW, using the deuterium dilution technique; the use of standard protocols for both data collection and data management; as well as the extent of 'north-south' collaboration between Europe and Africa. Others include development of a research protocol which had explicit emphasis on capacity building andtechnical co-operation; and the fact that the study sample size targets are being reached in most of the individual participating countries, and for the pooled data analysis which will be reported in subsequent publications.

The ROUND-IT Africa Study also had a number of weaknesses. Data generated are not representative of all African countries nor are they representative of the whole population of each participating country as the study was conducted in urban schools. As noted above, the problem of childhood obesity in Africa is localized largely in urban populations at present; therefore, the decision to obtain the study samples from urban areas was appropriate. In addition, the issue of representativeness of the sample is probably less important as methodological studies of this kind simply require a wide range of body fatness and PA data.

In conclusion, the ROUND-IT Africa Study is a multi-country research project that provides a foundation for improving future surveillance of both obesity and PA in children and adolescents across Africa, by a combination of (a) data pooling approach to validate surveillance methods, and (b) building obesity and NCD research capacity across 12 African countries. In the longer term, findings from ROUND-IT Africa Study and the research capacity which it has built should help to increase our understanding of the obesity epidemic in Africa, and this should guide future development of research and policy interventions designed to address childhood obesity in the continent.

This methodological report describes the rationale, procedures and standard operating procedures followed in the ROUND-IT study. Standardization of the protocol allows for data comparability and ROUND-IT provides a foundation for future public health interventions for childhood obesity in the African region.

Study strengths and limitations

In summary, the study's strengths and limitations are as follows:

- Use of standard reference methods for measurement of body composition (total body water by deuterium dilution) and PA (accelerometry)
- Cross-African 12-nation consortium which follows standard protocols for both data collection and management
- Samples recruited from cities across Africa, focusing on the areas most affected by the obesity and NCD pandemics

- Large study sample of 2000 children were recruited. Although we did not calculate the statistical power, the sample was assumed to have adequate statistical power (i.e. in conjunction with a nomogram chart proposed by Altman (1982), as cited by Altman (1991) for answering the main research questions (validity of BMI-for-age and questionnaire assessment of PA)
- Power may be limited for sub-group analyses (comparisons between North Africans vs East Africans vs Southern Africans vs Island populations)

Declarations

The principal investigators in each country were responsible for overseeing all aspects of the study. The role of the IAEA was to assess the overall progress as well as to assist and guide the study. Support on project research questions, methods, and data analyses were provided by IAEA consultants specializing in the necessary aspects as earlier stated.

Acknowledgements

The authors acknowledge the support of all the participants in the ROUND-IT Africa study. The role of the staff of the Division Exercise Science and Sports Medicine (ESSM), Department of Human Biology, Faculty of Health Sciences, University of Cape Town in facilitating the project is appreciated. The support received from from institutions and governments in the participating countries (e.g. South African study: Faculty of Health Sciences and Medical Research Council (MRC), and National Research Foundation (NRF): **Disclaimer;** Any opinion, findings and conclusions or recommendations expressed in this material are those of the author(s), and therefore the NRF and MRC do not accept any liability in this regard), is gratefully acknowledged. This work was supported by the International Atomic Energy Agency (Project RAF 6042).

References

Altman, D.G. (1991). *Practical Statistics for Medical Research* (p. 611). London: Chapman & Hall/CRC.

Alwan, A., Maclean, D.R., Riley, L.M, d'Espaignet, E.T., Mathers, C.D., Stevens, G.A. & Bettcher, D. (2010). Monitoring and surveillance of chronic non communicable diseases: Progress and capacity in high-burden countries. *The Lancet,* 376, 1861–1868.

Armstrong, M.E., Lambert, M.I., Sharwood, K.A. & Lambert, E.V. (2006). Obesity and overweight in South African primary school children: The Health of the Nation Study. *South African Medical Journal,* 96, 439–444.

Arvidsson, D., Fitch. M., Hudes, M.L., Tudor-Locke, C. & Fleming, S.E. (2011). Accelerometer response to physical activity intensity in normal-weight versus overweight African American children. *Journal of Physical Activity & Health*, 8,682-692.

Awotidebe, A., Monyeki, M.A., Moss, S.J., Strydom, G.L., Amstrong, M. & Kemper, H.C.G. (2016). Relationship of adiposity and health-related fitness on resting blood pressure of South African adolescents: the PAHL Study. *Journal of Human Hypertension*, 30, 245-251.

Benıtez-Porres, J., Lopez-Fernandez, I., Raya, J.F., Alvarez, Carnero, S., Alvero-Cruz, J.R. & Alvarez Carnero, E. (2016). Reliability and validity of the PAQ-C Questionnaire to assess physical activity in children. *Journal* of *School Health*, 86, 677-685.

Benjelloun, S. (2002). Nutrition transition in Morocco. *Public Health Nutrition*, 135-140.

Biddle, S.J., Gorely, T., Pearson, N. & Bull, F.C. (2011). An assessment of self-reported physical activity instruments in young people for population surveillance: Project ALPHA. *International Journal of Behavioral Nutrition and Physical Activity*, 8, 1. doi: 10.1186/1479-5868-8-1

Black, R.E., Victora, C.G., Walker, S.P., Bhutta, Z.A., Christian, P., de Onis. M., Ezzati M, Grantham-McGregor, S., Katz, J., Martorell, R., Uauy, R., Maternal and Child Nutrition Study Group (2013). Maternal and child undernutrition and overweight in low-income and middle-income countries. *The Lancet,* 382 (9890), 427-451.

Brown, M.L., Goldie, S., Draisma, G., Harford, J. & Lipscomb, J. (2006). Health service interventions for cancer control in developing countries. In D.T. Jamison, J.G. Breman, A.R. Measham, G. Alleyne, M. Claeson, D.B. Evans, P. Jha, A. Mills & P. Musgrove (Eds.). *Disease Control Priorities in Developing Countries* (2nd ed.) (pp. 569-589). New York: Oxford University Press.

Craig, E., Reilly, J. & Bland, R. (2013). Body fatness or anthropometry for assessment of unhealthy weight status? Comparison between methods in South African children and adolescents. *Public Health Nutrition,* 16(11), 2005-13. doi: 10.1017/S1368980012004338.

Crocker, P.R., Bailey, D.A., Faulkner, R.A., Kowalski, K.C. & McGrath, R. (1997). Measuring general levels of physical activity: Preliminary evidence for the Physical Activity Questionnaire for older children. *Medicine & Science* in *Sports & Exercise,* 29(10), 1344-1349.

de Onis, M., Onyango, A. W., Borghi, E., Siyam, A., Nishida, C. & Siekmann, J. (2007). Development of a WHO growth reference for school-aged children and adolescents. *Bulletin of World Health Organisation*, 85(9), 660-667.

de Villiers, A., Steyn, N.P., Draper, C.E., Fourie, J.M., Barkhuizen, G., Lombard, C.J., Dalais, L., Abrahams, Z. & Lambert, E.V. (2012). "HealthKick": Formative assessment of the health environment in low-resource primary schools in the Western Cape Province of South Africa. *BMC Public Health,* 12, 934.

Dinesh, J. & Patty, F. (2012). Actigraph and actical physical activity monitors: A peek under the hood. *Medicine & Science* in *Sports & Exercise*, 44, Suppl 1, S86–S89.

Gillespie, S., Haddad, L., Mannar, V., Menon, P. & Nisbett, N. (2013). The politics of reducing malnutrition: Building commitment and accelerating progress. *The Lancet,* 382 (9891), 552-569.

Goran, M.I., Nagy, T.R., Treuth, M.T., Trowbridge, C., Dezenberg, C., McGloin, A. & Gower, B.A. (1997). Visceral fat in Caucasian and African-American pre-pubertal children. *American Journal of Clinical Nutrition,* 65, 1703–1708.

Guthold, R., Cowan, M.J., Autenrieth, C.S., Kann, L. & Riley, L.M. (2010). Physical activity and sedentary behavior among schoolchildren: A 34-country comparison. *The Journal of Pediatrics*, 157(1), 43-49.

Hallal, P.C., Reichert, F.F., Clark, V.L., Cordeira, K.L., Menezes, A.M., Eaton. S., Ekelund, U. & Wells, J.C. (2013). Energy expenditure compared to physical activity measured by accelerometry and self-report in adolescents: A validation study. *PLoS One,* 4, 8(11), e77036.

Hanevold, C., Waller, J., Daniels, S., Portman, R. & Sorof, J. (2004). The effects of obesity, gender, and ethnic group on left ventricular hypertrophy and geometry in hypertensive children: A collaborative study of the International Pediatric Hypertension Association. *Pediatrics,*113, 328–333.

Heil, D.P., Brage, S. & Rothney, M.P. (2012). Modeling physical activity outcomes from wearable monitors. *Medicine & Science in Sports & Exercise*, 44, S50–S60.

International Atomic Energy Agency (2010). *Human Health Series (HHS). No.12*. Vienna, Austria: International Atomic Energy Agency.

Javed, A., Jumean, M., Murad, M.H., Okorodudu, D., Kumar, S., Somers, V.K., Sochor, O. & Lopez-Jimenez, F. (2015). Diagnostic performance of body mass index to identify obesity as defined by body adiposity in children and adolescents: A systematic review and meta-analysis. *Pediatric Obesity*, 10(3), 234-244.

Kamoun, M., Hajem, S., Imen, S., Achour, N. & Slimane, H. (2008). Prevalence of obesity and overweight in Tunisia on 2001. *Tunisia Medicine,* 86(7), 649-652.

Keetile, M., Navaneetham, K. & Letamo, G. (2015). Patterns and determinants of hypertension in Botswana. *Journal of Public Health*, 23(5), 311-318.

Kowalski, K., Crocker, P. & Donen, R. (2004). *The Physical Activity Questionnaire for Older Children (PAQ-C) and Adolescents (PAQ-A) Manual*. Canada: University of Saskatchewan.

Kuba, V.M., Leone, C. & Damiani, D. (2013). Is waist-to-height ratio a useful indicator of cardio-metabolic risk in 6-10-year-old children? *BMC Pediatrics*, 13, 91.

Lauritsen, J.M. & Bruus, M. (2008). *Epidata Entry. A Comprehensive Tool for Validated Entry and Documentation of Data*. Odense Denmark: The EpiData Association.

Li, W.C., Chen, I.C., Chang, Y.C., Loke, S.S., Wang, S.H. & Hsiao, K.Y. (2013). Waist-to-height ratio, waist circumference, and body mass index as indices of cardiometabolic risk among Taiwanese adults. *European Journal of Nutrition*. 52(1), 57–65.

Liu, A., Byrne, N.M., Kagawa, M., Ma, G., Poh, B.K., Ismail, M.N. & Hills, A.P. (2011). Ethnic differences in the relationship between body mass index and percentage body fat among Asian children from different backgrounds. *British Journal of Nutrition*, 106(9), 1390-1397.

Lohman, T.G. (1986). Applicability of body composition techniques and constants for children and youths. *Exercise and Sports Science Reviews*, 14, 325-357.

Lohman, T.G. (1992). *Advances in Body Composition Assessment* (pp. 65–77). Champaign, IL.: Human Kinetics publishers.

Luke, A., Bovet, P., Forrester, T.E., Lambert, E.V., Plange-Rhule, J., Dugas, L.R., Durazo-Arvizu, R.A., Kroff, J., Richie, W.N. & Schoeller, D.A. (2013). Prediction of fat-free mass using bioelectrical impedance analysis in young adults from five populations of African origin. *European Journal of Clinical Nutrition,* 67(9), 956–960.

Luke, A., Durazo-Arvizu, R., Rotimi, C., Prewitt, T.E., Forrester, T., Wilks, R., Gunbiyi, O.J., Schoeller, D.A., McGee, D. & Cooper, R.S. (1997). Relation between body mass index and body fat in black population samples from Nigeria, Jamaica, and the United States. *American Journal of Epidemiology*, 145, 620–628.

Mciza, Z.J., Goedecke, J.H. & Lambert, E.V. (2011). Intra-familial and ethnic effects on attitudinal and perceptual body image: A cohort of South African mother-daughter dyads. *BMC Public Health,* 6;11, 433. doi:10.1186/1471-2458-11-433.

Micklesfield, L.K., Pedro, T.M., Kahn, K., Kinsman, J., Pettifor, J.M., Tollman, S. & Norris, S.A. (2014). Physical activity and sedentary behavior among adolescents in rural South Africa: Levels, patterns and correlates. *BMC Public Health*, 14, 40. doi: 10.1186/1471-2458-14-40.

Mokha, J.S., Srinivasan, S.R., Dasmahapatra, P., Fernandez, C., Chen, W., Xu, J. & Berenson, G.S. (2010). Utility of waist-to-height ratio in assessing the status of central obesity and related cardiometabolic risk profile among normal weight and overweight/obese children: The Bogalusa Heart Study. *BMC Pediatric*, 11; 10, 73. doi: 10.1186/1471-2431-10-73.

Monyeki, M.A., Koppes, L.L.J., Kemper, H.C.G., Monyeki, K.D., Toriola, A.L., Pienaar, A.E. & Twisk, J.W.R. (2005). Body composition and physical fitness of undernourished South African rural primary school children. *European Journal of Clinical Nutrition,* 59, 877-883.

Monyeki, A., Neetens, R., Moss, H. & Twisk, J. (2012). The relationship between body composition and physical fitness in 14 years old adolescents residing within the Tlokwe local municipality, South Africa: The PAHL Study. *BMC Public Health*, 12, 374.

Moore, J.B., Hanes, J.C., Barbeau, P., Gutin, B., Trevino, R.P. & Yin, Z. (2007). Validation of the Physical Activity Questionnaire for older children in children of different races. *Pediatric Exercise Science,* 19, 6–19.

Muthuri, S.K., Wachira, L-J,M., Leblanc, A.G., Francis, C.E., Sampson, M. & Onywera, T. (2014). Temporal trends and correlates of physical activity, sedentary behaviour, and physical fitness among school-aged children in Sub-Saharan Africa: A systematic review. *International Journal of Environmental Research & Public Health,* 11(3), 3327–3359.

Muthuri, S.K, Wachira, L-J.M, Onywera, V.O. & Tremblay M.S. (2014). Correlates of objectively measured overweight/obesity and physical activity in Kenyan school children: Results from ISCOLE-Kenya. *BMC Public Health*, 14, 436. http://www.biomedcentral.com/1471-2458/14/436.

National High Blood Pressure Education Program Working Group on High Blood Pressure in Children and Adolescents (2004). The fourth report on the diagnosis, evaluation, and treatment of high blood pressure in children and adolescents. *Pediatrics,* 114, 555-5576.

National High Blood Pressure Education Program (1996). Task Force Report on High Blood Pressure in Children and Adolescents: A working group report from the National High Blood Pressure Education Program. National High Blood Pressure Education Program Working Group on Hypertension Control in Children and Adolescents. *Pediatrics,* 98, 649-658.

NHLBI Obesity Education Initiative (2000). *The Practical Guide to the Identification, Evaluation, and Treatment of Overweight and Obesity in Adults*. Rockville: National Institutes of Health.

Nightingale, C.M., Rudnicka, A.R., Owen, C.G., Cook, D.G. & Whincup, P.H. (2011). Patterns of body size and adiposity among UK children of South Asian, black African-Caribbean and white European origin: Child Heart and Health Study in England (CHASE study). *International Journal of Epidemiology,* 40, 33–44.

Oulamara, H., Agli, A.N. & Frelut, M.L. (2009). Changes in the prevalence of overweight, obesity and thinness in Algerian children between 2001 and 2006. *International Journal* of *Pediatric Obesity*. 4, 411–413.

Oyeyemi, A., Moss, J.S., Monyeki A.M. & Kruger, S.H. (2016). Measurement of physical activity in urban and rural South African adults: A comparison of two self-report methods. *BMC Public Health*, 16:1004. DOI 10.1186/s12889-016-3693-3696.

Parsons, T.J., Power, C., Logan, S. & Summerbell, C.D. (1999). Childhood predictors of adult obesity: A systematic review. *International Journal of Obesity*, 23, Suppl 8, S1-S107.

Plasqui, G., Bonomi, A.G. & Westerterp, K.R. (2013). Daily physical activity assessment with accelerometers: New insights and validation studies. *Obesity Review,* 14(6), 451-462.

Popkin, B.M. (1998). The nutrition transition and its health implications in lower-income countries. *Public Health Nutrition,* 1, 5–21.

Prins, M., Hawkesworth, S., Wright, A., Fulford, A.J.C., Jarjou, L.M.A., Prentice, A.M. & Moore, S.E. (2008). Using of bioelectrical impendance analysis to assess body composition in rural Gambian children. *European Journal of Clinical Nutrition,* 62, 1065–1074.

Raj, M. & Krishnakumar, R. (2013). Hypertension in children and adolescents: Epidemiology and pathogenesis. *The Indian Journal of Pediatrics,* 80, Suppl 1, S71-76.

Reddy, S.P., James, S., Sewpaul. R., Koopman, F., Funani, N.I., Sifunda, S., Masuka, J.J., Kambaran, P. & Omardien, R.G. (2010). *Umthente Uhlaba Usamila – The Second South African Youth Risk Behaviour Survey 2008*. Cape Town: South African Medical Research Council. http://www.mrc.ac.za/healthpromotion/ healthpromotion.htm.

Reilly, J.J. (2006). Diagnostic accuracy of the BMI for age in paediatrics. *International Journal of Obesity,* 30, 595–597.

Reilly, J.J. (2010). Low levels of objectively measured physical activity in preschoolers in child care. *Medicine and Science in Sports Exercise*, 42(3), 502-507.

Reilly, J.J. & Kelly, J. (2010). Long-term impact of overweight and obesity in childhood and adolescence on morbidity and premature mortality in adulthood: Systematic review. *International Journal of Obesity*, 35, 891–898.

Reilly, J.J., Dorosty, A.R., Ghomizadeh, N.M., Sherriff, A., Wells, J.C. & Ness, A.R. (2010). Comparison of waist circumference percentiles versus body mass index percentiles for diagnosis of obesity in a large cohort of children. *International Journal of Pediatric Obesity*, 5,151–156.

Rich, C., Griffiths, L.J. & Dezateux, C. (2012). Seasonal variation in accelerometer-determined sedentary behaviour and physical activity in children: A review. *International Journal of Behavioral Nutrition and Physical Activity*, 30 (9), 49.

Rich, C., Geraci, M., Griffiths, L., Sera, F., Dezateux, C. & Cortina-Borja, M. (2013). Quality control methods in accelerometer data processing: Defining minimum wear time. *PLoS ONE*, 8, e67206.

Rowlands, A.V. & Eston, R.G. (2007). The measurement and interpretation of children's physical activity. *Journal of Sport Science Medicine*, 6, 270–276.

Sasaki, J.E., John, D. & Freedson, P.S. (2011). Validation and comparison of ActiGraph activity monitors. *Journal of Science and Medicine in Sport*, 14(5), 411–416.

Schoeller, D.A., Van Santen, E., Paterson, D.W., Dietz, W., Jaspan, J. & Klein, P.D. (1980). Total body water measurement in humans with 18O and 2H labeled water. *American Journal of Clinical Nutrition*, 33, 2688–2693.

Shah, N.R. & Braverman, E.R. (2012). Measuring adiposity in patients: the utility of body mass index (BMI), percent body fat, and leptin. *PLoS One,* 7(4), e33308.

Shisana, O., Labadarios, D., Rehle, T., Simbayi, L., Zuma, K., Dhansay, A., Reddy, P., Parker, W., Hoosain, E., Naidoo, P., Hongoro, C., Mchiza, Z., Steyn, N.P., Dwane, N., Makoae, M., Maluleke, T., Ramlagan, S., Zungu, N., Evans, M.G., Jacobs, L., Faber. M. & SANHANES-1 Team (2013). South African National Health and Nutrition Examination Survey (SANHANES-1). Cape Town: HSRC Press.

Stergiou, G.S., Yiannes, N.G. & Rarra, V.C. (2006). Validation of the Omron 705 IT oscillo-metric device for home blood pressure measurement in children and adolescents: The Arsakion School Study. *Blood Pressure Monitor*, 11, 229-234.

Tremblay, M.S., Gray, C.E., Akinroye, K., Harrington, D.M., Katzmarzyk, P.T., Lambert, E.V., Liukkonen, J., Maddison, R., Ocansey, R.T., Onywera, V.O., Prista, A., Reilly, J.J., Rodríguez, Martínez, M.P., Sarmiento Duenas, O.L., Standage, M. & Tomkinson. G. (2014). Physical activity of children: A global matrix of grades comparing 15 countries. *Journal of Physical Activity and Health*, 11, Supp 1, S113-S125.

Troiano, R.P., Berrigan, D., Dodd, K.W., Masse, L.C., Tilert, T. & McDowell, M. (2008). Physical activity in the United States measured by accelerometer. *Medicine & Science* in *Sports & Exercise*, 40,181–188.

Tudor-Locke, C., Craig, C.L., Beets, M.W., Belton, S., Cardon, G.M. & Duncan S. (2011). How many steps/day are enough? For children and adolescents. *International Journal of Behavioral Nutrition and Physical Activity,* 8, 78.

Vorster, H.H., Kruger, A. & Margetts, B.M. (2011). The nutrition transition in Africa: Can it be steered into a more positive direction? *Nutrients*, 3, 429-441.

Wang, J.J., Baranowski, T., Lau, W.P., Chen, T.A. & Pitkethly, A.J. (2016). Validation of the Physical Activity Questionnaire for older children (PAQ-C) among Chinese children. *Biomedical* and *Environmental* Sciences, 29(3), 177-186.

Wikepedia. https://en.wikipedia.org/wiki/Tanzania (accessed on the 28 May 2019).

Wells, J.C.K. & Fewtrell, M.S. (2006). Measuring body composition. *Archives* of *Disease in Childhood*, 91(7), 612–617.

Williams, D.P., Going, M.S., Scott, B., Lohman, T.G., Harsha, D.W., Snnivasan, S.R., Lany, S. Webber, L.S. & Berenson, G.S. (1992). Body fatness and risk for elevated blood pressure, total cholesterol, and serum lipoprotein ratios in children and adolescents. *American Journal of Public Health*, 82(4), 527.

World Health Organization (2007). WHO *Child Growth Standards: Head Circumference-for-age, Arm Circumference-for-age, Triceps Skinfold-for-age and Subscapular Skinfold-for-age: Methods and Development*. Geneva: WHO.

World Health Organization (2008). *Training Course on Child Growth Assessment*. Geneva: WHO.

World Health Organization (2011). *WHO child growth standards: Length/height-for-age, Weight-for-age, Weight-for-length, Weight-for-height and Body Mass Index-for-age: Methods and Development.* Geneva: WHO.

World Health Organization (2011). *Waist Circumference and Waist-Hip Ratio: Report of a WHO Expert Consultation. Geneva, 8–11 December 2008*. Geneva: WHO.

World Health Organization (WHO) (2014). Global Status Report on Non-communicable disease. http://www.who.int/nmh/publications/ncd_report_full_en.pdf).

World Health Organization (2016). *Report of the Commission on Ending Childhood Obesity*. Geneva: WHO.

List of abreviations

BMI: body mass index
ROUND-IT: Reducing Obesity Using Nuclear Techniques to Design Interventions in Africa
WHO: World Health Organization
PAQ-C: Physical Activity Questionnaire for Children
NCD's: Non-Communicable Diseases
PA: Physical Activity
TBW: Total Body Water
IAEA: International Atomic Energy Agency
D_2O: Deuterium Oxide
SES: Socioeconomic Status
ESSM: Exercise Science and Sports Medicine
PANES: Physical Activity Neighborhood Environment Survey
SHAPES: School Health Environment Survey
HIV/AIDS: Human Immunodeficiency Virus Infection /Acquired Immune Deficiency Syndrome
SD: Standard Deviation
BP: Blood Pressure
SBP: Systolic Blood Pressure
MVPA: Moderate Vigorous Physical Activity
FTIR: Fourier Transform Infrared Spectrometry
FFM: Fat Free Mass
FM: Fat Mass
SOP: Standard Operating Procedures
QA-QC: Quality assurance and Quality Control
ROC: Receiver Operating Characteristics

Authors' contributions (Identified by their initials)

1. Substantial contributions to:
a. Conception and design: all authors.
b. Data acquisition: AELH, TA, AA, VDA, AD, NIJ, GHL, KDM, TM, MAM, DN, HLN, AFS.
c. Data analysis and interpretation: led by SMAS, AELH, AD, with contributions from all authors.
2. Involvement in:
a. Drafting protocol manuscript: led by AELH, MAM, JJR, TM, CL, NIJ, GHL.
b. Revising protocol manuscript for important intellectual content: all authors.
3. Approval for publication: all authors.
4. Agreed to be accountable for publication: all authors.
All authors read and approved the final manuscript.
